# Autonomous Implants

**DOI:** 10.1002/adma.202510702

**Published:** 2025-10-13

**Authors:** Jagan Mohan Dodda, Marcel F. Kunrath, Ling Zhou Zhao, Mahdi Bodaghi, Armin Yousefi, Nureddin Ashammakhi

**Affiliations:** ^1^ New Technologies–Research Centre (NTC) University of West Bohemia University 8 Pilsen 301 00 Czech Republic; ^2^ Department of Biomaterials Institute of Clinical Sciences Sahlgrenska Academy University of Gothenburg Sweden; ^3^ School of Health and Life Sciences Dentistry Department Pontifical Catholic University of Rio Grande do Sul (PUCRS) Porto Alegre Brazil; ^4^ Department of Stomatology FMMU Beijing 100142 China; ^5^ Department of Engineering School of Science and Technology Nottingham Trent University Nottingham NG11 8NS UK; ^6^ Institute for Quantitative Health Science and Engineering (IQ) College of Human Medicine Michigan State University East Lansing MI 48824 USA

**Keywords:** autonomous implant, self‐actuating, self‐aware, self‐forming, self‐healing, self‐powering, self‐regeneration

## Abstract

With the increasing aging population, more implants are being used in the body. However, these implants often fail, and they suffer from a lack of integration or proper function. Efforts have been made to generate implants by employing bioactive materials, adding cellular components, and integrating smart characteristics such as self‐healing properties. The vision is, however, to develop an implant that can mimic native tissues in their way of doing things, responding to challenges and remodeling. Such automated implants require the integration of advances made in various fields of science. Although early, there are good steps taken in this way, e.g., the development of stimuli‐responsive, self‐powering, self‐actuating, self‐healing, self‐regenerating, self‐aware implants. Attempts to combine more than one smart property into these implants are still at the beginning, e.g., the integration of such special characteristics requires a new set of skills and thinking, which presents new challenges that warrant exploration and investment. Such an implant evolution is expected to be in stages, where the first implant will be able to communicate with doctors and hospitals; then, in the next stage, with patients, and later, they will be independent, sense any disturbances and aberrations from normal early on, and correct themselves before damage becomes irreversible. This forward‐looking review looks at the research done thus far in this direction and efforts to assemble individual aspects of smart implants into multifunctional implants in the future. The stages of material, in vitro, and in vivo testing and clinical application, if any, are critically reviewed. In addition, the challenges facing the development of autonomous smart implants are discussed, and research directions and ideas are suggested.

## Graphical abstract

An ideal implant should mimic native tissues such that it can integrate, sense, heal, and continue to function, i.e., be autonomous. Although early, there are good steps taken in this way, e.g., the development of stimuli‐responsive, self‐powering, self‐actuating, self‐healing, self‐regenerating, and self‐aware implants. This forward‐looking review outlines the research done thus far in this direction and efforts to assemble individual aspects of smart implants into a multifunctional implant in the future.

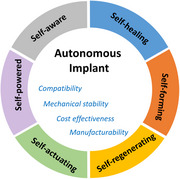

## 1 Introduction

Implants are being increasingly inserted into the body to repair, replace or help regenerate damaged or lost tissues and organs.^[^ ^1^, ^2^ ^]^ Biomaterials have evolved to be more biocompatible and smarter. First, implants were stable and bioinert, and successful examples included the introduction of hip prostheses to the clinic by the end of the 1960s.^[^ ^3^ ^]^ The discovery of bioactive materials such as bioactive glass led us to consider a new concept in which an implant can bond to tissues.^[^ ^4^ ^]^ Later, multifunctional biomaterials were developed, and functions such as drug release properties were added to implants to enable them to achieve faster and better healing^[^ ^5^, ^6^ ^]^ or treat complications such as infection.^[^ ^7^, ^8^ ^]^ With the advent of tissue engineering in the late 1980s,^[^ ^9^, ^10^ ^]^ it became possible to construct tissues ex vivo^[^ ^11^ ^]^ or in situ.^[^ ^12^ ^]^ Most recently, the use of three‐dimensional bioprinting (3DBP) has enabled better control of cell and additive locations in implants.^[^ ^13^ ^]^ Furthermore, the integration of advances made in other fields of science has enabled the integration of sensors into implants that can help pick up changes in the implants themselves or in their surroundings before problems become irreversible; thus, appropriate intervention can be instituted.^[^ ^14^, ^15^ ^]^ This makes implants smarter and has some characteristics that emulate native tissues. More technologies are becoming available to add more biomimetics in nature. The vision is to develop autonomous implants in the future, which are smarter and have the characteristics of “living” tissues in the body. To realize this vision, such implants need to have different autonomy (autonomousness) properties, such as self‐awareness,^[^ ^16^, ^17^ ^]^ self‐regeneration,^[^ ^18^, ^19^ ^]^ self‐powering,^[^ ^20^ ^]^ self‐actuation,^[^ ^21^ ^]^ etc. Recently, studies have been published that have advanced each of these components (properties) separately (independent of the other).^[^ ^22^ ^]^

**Figure 1:**
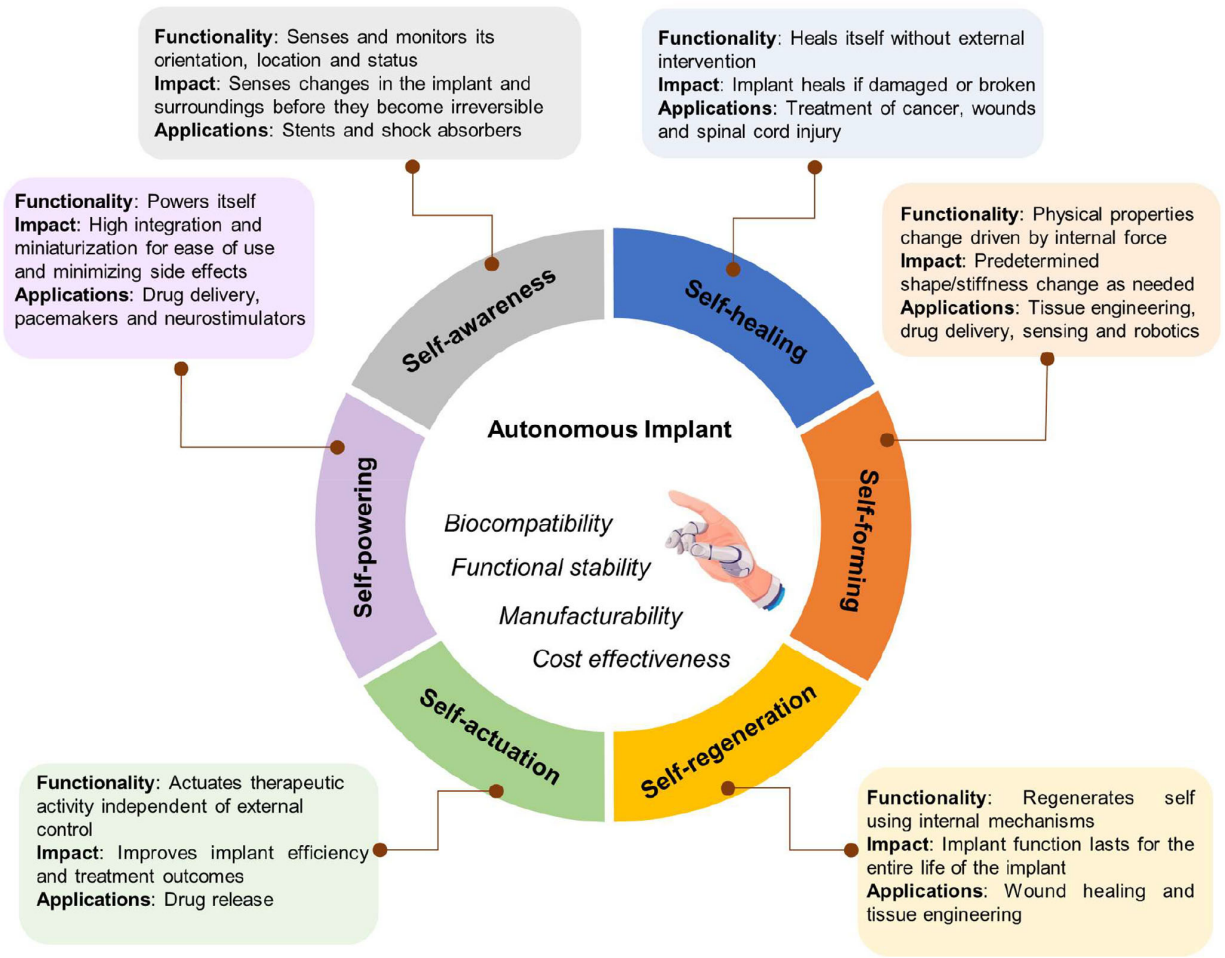
Concept of visionary autonomous implant of the future. A schematic showing the components of the autonomous implant discussed in the review and their specific features and applications, which will enable the realization of autonomous implants.

With advances in basic science, including chemistry and biology; engineering, including chemical, biomedical, and information technology; and integrated systems for clinical applications, hybrid implants are being developed to produce devices that can mimic native tissues in health and disease.^[^ ^23^, ^24^ ^]^ Such implants are autonomous and function independently without the need for external intervention. To this end, such a new generation of implants has essentially been multifunctional in nature. It needs to be self‐aware,^[^ ^16^ ^]^ sensitive to self and surroundings,^[^ ^22^, ^25^, ^26^ ^]^ self‐actuating where action is needed,^[^ ^27^ ^]^ self‐healing if it sustains damage^[^ ^28^ ^]^ and communicating^[^ ^14^ ^]^ to alert the organism for further actions (**Figure** **1**). With the advanced tools we currently have, communication can also rely on the outside environment to rely on data from the implant and its environment, and receive external commands. Developing that concept and designing autoimplants is a challenging task, as technologies are fragmented and require appropriate integration by multidisciplinary knowledgeable teams who can carry this vision forward. The other challenge in developing these autonomous implants is the type of biomaterials that need to be utilized to produce such implants and make them biocompatible with human tissues. Although advanced fabrication techniques, such as 3D^[^ ^29^ ^]^ and four‐dimensional (4D)^[^ ^30^ ^]^ printing, as well as self‐powering systems,^[^ ^31^, ^32^ ^]^ nanogenerators and devices,^[^ ^33^, ^34^ ^]^ have enabled the pursuit of new directions or paths for designing autoimplants, the large number of medical problems we have today need continuous integration of technological advances, which will enable us to engineer autonomous implants.

To our knowledge, no study has yet combined all these properties. Therefore, we compiled this review to develop a vision for combining these properties toward the development of autonomous implants of the future. This paper discusses the individual technologies needed to produce such multifunctional devices, including key advances in the design and development of self‐aware, self‐sensing, self‐healing, self‐actuating, self‐powered implants. We reviewed advances in these self‐functioning implants in various sections, accounting for their concept, mechanism, and in vitro and in vivo studies, and searched the literature for any reported clinical trials. In addition, their characterization methods, including animal experiments and potential clinical applications, are discussed. Furthermore, current challenges and ways to address them are highlighted, and directions for future research are introduced.

## 2 Properties required to Develop Autonomous Implants

Autonomous implants in the future are required to possess certain properties and functionalities, which include some common general and specific ones discussed in the following sections.

### 2.1 General Requirements of a Smart Implant

#### 2.1.1 Mechanical Requirements

A successful implant should have mechanical properties that match those of the target tissue. Since healing is a dynamic process in which the mechanical properties of the newly formed and healed tissue change continuously, the implant properties should ideally match these changes. Matching mechanical properties can impact tissue reactions, e.g., those in the brain,^[^ ^35^ ^]^ where neural implants with ultrathin polyimide substrates and silicon shuttles that have mechanical properties comparable to those of brain tissue are more biocompatible. Compared with other metal‐ or silicon‐based interfaces, which have higher elastic moduli than do biological tissues,^[^ ^36^ ^]^ the low bending stiffness of these implants results in reduced inflammatory response trauma, whereas bone implants should have mechanical properties closer to those of the bone.^[^ ^37^, ^38^, ^39^ ^]^ For example, orthopedic implants need to be strong enough to withstand external forces and promote long‐term stability. However, the use of rigid implants may lead to stress shielding after implantation, which may lead in turn to resorption of the adjacent bone tissue and an increased risk of loosening and even fracture.^[^ ^40^ ^]^

In normal situations, tissues may also change with age and the remodeling process; thus, implants should be able to cope with these changes. Therefore, this is a requirement that should ideally be met by future advanced implants and be an essential component of future autonomous implants that can undergo changes as needed. On the other hand, implants in the body are exposed to continuous challenges, especially skeletal implants; thus, initial and sustained mechanical properties should be defined. Furthermore, implant ductility is another important requirement for implant contouring and shaping. The implant should have high mechanical strength, fatigue strength, and compression strength to prevent fractures and improve stability. The stress transfer between the bone and the implant plays a crucial role in synchronizing the implant with the bone or internal tissues to protect them from severe internal damage. The implant should have good elasticity to ensure a more uniform stress distribution at the implant and to minimize the relative movement at the interface between the implant and the bone. Similarly, implants exposed to harsh environments created by inflammation,^[^ ^41^ ^]^ acids,^[^ ^42^, ^43^ ^]^ or aqueous paths (such as saliva)^[^ ^44^, ^45^ ^]^ can greatly affect implant stability and may lead to failure.^[^ ^46^ ^]^

The mechanical stability of smart implants, such as flexible and stretchable electronics, is of utmost importance. The reliable performance of flexible electronic devices depends on the stability of key electrical properties, such as resistance, capacitance, and response speed, even under mechanical deformation.^[^ ^47^, ^48^, ^49^, ^50^ ^]^ However, throughout their operational lifespan, these devices are subjected to complex and repeated strains that can lead to material fatigue, failure, or signal degradation. Such mechanical challenges are particularly pronounced during integration with dynamic biological substrates, including human skin and internal organs, or when these substrates are incorporated into wearable textile systems.^[^ ^47^, ^48^, ^49^, ^50^ ^]^ Fortunately, the long‐term functionality of many of these implants in the body has already been demonstrated.^[^ ^51^ ^]^ The durability of flexible electronics under substantial tensile loading is largely governed by the initiation and propagation of cracks, which extend and ultimately compromise the structural integrity and electrical performance of the device.^[^ ^52^, ^53^, ^54^, ^55^ ^]^ Another type of failure is bending. Most of these types of implants are completely attached to muscles, so by deformation of the muscles, the implants are subjected to bending.^[^ ^47^ ^]^ If the deformation is too large, it leads to permanent deformation in the device. Under bending conditions, the device is subjected to both tensile and compression stresses. Brittle components in flexible electronic devices are prone to failure because of stress concentrations under bending conditions. This failure mode may also occur at interconnect regions. Additionally, bending‐induced stresses can distort or destabilize structural features such as microbending patterns and 3D elements, ultimately degrading device performance.^[^ ^47^, ^51^, ^56^, ^57^ ^]^ Additionally, impact force can cause serious damage to implants, regardless of their nature, autonomous or conventional. Flexible engineering devices are susceptible to unexpected impacts or collisions, which may result in mechanical failure.^[^ ^47^ ^]^ A well‐recognized trade‐off exists between mechanical robustness and functional performance in flexible electronics. Specifically, efforts to improve resistance to external mechanical shocks often come at the expense of sensitivity, monitoring accuracy, flexibility, and stretchability.^[^ ^47^, ^58^, ^59^, ^60^, ^61^, ^62^ ^]^

#### 2.1.2 Chemical Requirements

The implants should degrade or be stable, as the clinical indications may dictate. For example, a hip prosthesis is required to stay permanently, which a bone fixation plate may not, and hence, degradability should be tailored accordingly. Surface chemistry is an important determinant of molecular and cell attachment and consequent immune reactions that may lead to regeneration, e.g., by polarizing macrophages toward the M2 phenotype or toward an inflammatory reaction that may ultimately lead to the loss of the implant. In addition, the chemistry of the surface can be manipulated by fading different functionalities that can selectively favor the attachment of certain molecules or cells. In addition, the matrix of the implants can include and controllably release certain agents that may control implant function and the healing of fractures that may occur during impact or induce cellular functions or tissue regeneration. Implants are exposed to body fluids or factors that lead to their corrosion, disintegration or degradation, such as wear debris,^[^ ^63^ ^]^ colloidal organometallic complexes,^[^ ^64^ ^]^ ^65^ ^]^ inorganic salts,^[^ ^66^ ^]^ oxides,^[^ ^66^ ^]^ or metallic ions.^[^ ^67^ ^]^ Studies have shown that long‐term use of metallic implants,^[^ ^68^, ^69^ ^]^ toxic products such as nickel (Ni), copper (Co) and chromium (Cr)^[^ ^70^, ^71^ ^]^ may be released into the body during the corrosion process.^[^ ^72^, ^73^ ^]^ These factors should be taken into consideration when designing an implant, selecting its materials, and deciding on its target chemical properties. Released parts or metabolites should not have major toxicity or vital organ accumulation. Although biodegradable magnesium (Mg)‐based implants are promising for orthopedic implants, the release of hydrogen leads to problems such as inflammation and pain.^[^ ^74^, ^75^, ^76^ ^]^

#### 2.1.3 Biological Requirements

The implant should be tolerated by the body. Tissue reactions to such implants should be controllable and not interfere with implant function. Biocompatibility depends on factors such as the material of the implant, corrosion resistance, degradation, release of cytotoxic products, surface properties, and mechanical characteristics.^[^ ^77^, ^78^, ^79^ ^]^ Alterations in these attributes can affect biocompatibility; e.g., increased surface roughness leads to an increase in the surface area of the implant and thereby improves cell attachment, implantation, and integration. Implants made of relatively bioinert materials, such as metal alloys, induce limited tissue responses. Bioactive materials such as bioactive glass induce the formation of an appetite layer that helps the bonding of the implant to the surrounding tissues, whereas biodegradable implants such as polymers induce an inflammatory response, which is a part of their elimination process. Stimuli‐responsive materials can be controlled to behave in the desired way, e.g., they can undergo degradation and change their shape or release their load according to need. The biological response can also be controlled by the implant material, e.g., to induce a defined immune response, such as proregenerative M2 macrophage differentiation and reduced proinflammatory M1 macrophage formation. Hence, controlled implant behavior and related biocompatibility represent important aspects of autonomous implants and can be achieved by controlling various factors contributing to their biological behavior in a dynamic manner.

### 2.2 Specific Requirements of Autonomous Implants

In addition to the properties and attributes of smart implants in the future, autonomous implants also need to have self‐awareness, self‐actuation, self‐forming, and self‐regeneration, the details of which are discussed in the following sections. To date, only the individual properties of self‐awareness, self‐healing, self‐forming or self‐healing are known, with only a few cases where implants combine two or more of these attributes, such as spinal fusion cage implants with self‐sensing and self‐powering capabilities,^[^ ^16^ ^]^ cardiovascular stents and shock absorbers with self‐sensing and self‐powering capabilities.^[^ ^17^ ^]^ The possibility of compiling these individual self‐performing implants or autonomous components into one multifunctional autonomous implant is the gateway for new implantable technologies in the future. In the following sections, we discuss the advances made in these individual fields. The number and type of components required are determined by the type of clinical application, which guides the implant features needed. In addition, the implant should be sterilized and monitored via commonly used imaging techniques, such as X‐rays and computed tomography (CT) scans.

#### 2.2.1 Self‐Healing

Self‐healing of an implant is its ability to heal itself after sustaining damage due to physical or chemical stresses resulting from internal or external causes. The concept of self‐healing was first introduced in 1969 as “self‐healing of cracks” observed in polyvinyl acetate.^[^ ^80^ ^]^ Later, the concept was associated with polymer composites in 2001.^[^ ^81^ ^]^ Recently, the self‐healing concept in the field of medicine reached its apex by developing several types of biomaterials with self‐healing properties.^[^ ^82^ ^]^ The purpose of using self‐healing implants is the extension of their lifetime,^[^ ^83^ ^]^ and to avoid inadvertent damage to tissues that may result from implant failure or fracture. Although self‐healing materials can exist in different forms, studies have investigated self‐healing materials used as coatings of implants or as hydrogels (Table 2).

##### Mechanism

**Figure 2:**
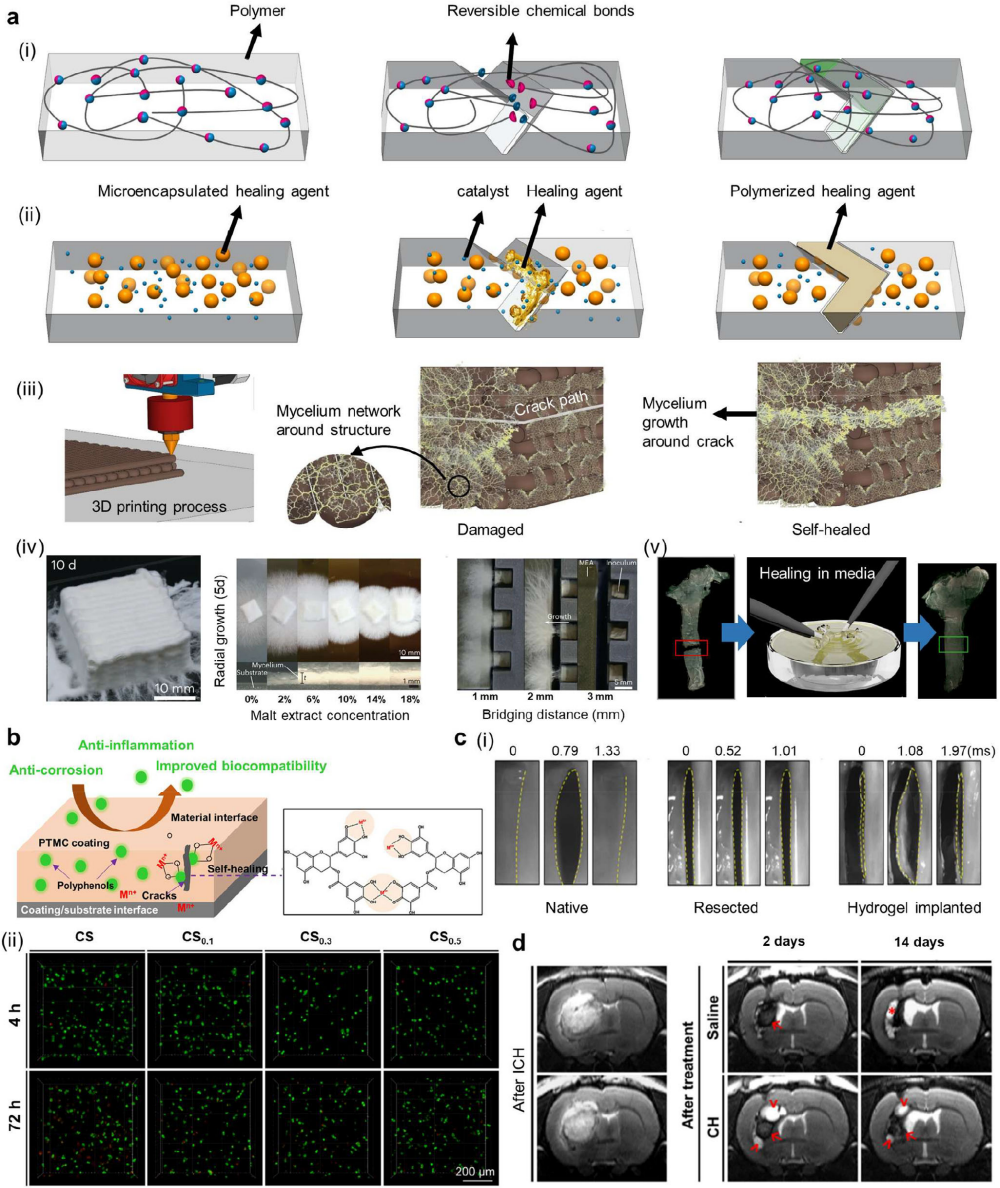
Schematic illustration: a) Mechanisms of intrinsic (i) and extrinsic (ii) self‐healing. i) Intrinsic approaches include materials that are based on bonding (covalent or noncovalent, imine or disulfide), and ii) extrinsic approaches include 1) microencapsulation of healing agents (fillers) that activate the healing process. 2) Microorganism‐based self‐healing methods, viz., fungal mycelial growth‐based methods, and 3) cellulose‐producing bacteria‐based methods. The growth pattern of mycelia inoculated for five days with bridge gaps in agar gels. Three‐dimensional (3D)‐printed mycelium‐based grids‐initial state and ten days of incubation, showing clear growth of mycelia between printed hydrogel filaments. 4) Image of the printed bronchial structure after damage, which was placed into the medium to regrow cellulose fibers by G. xylinus bacteria in the printed structure and self‐healed structure after three days of incubation).^[^ ^102^ ^]^ b) Material studies: Coating implants: self‐healing coating by polyphenols on the surface of Mg implants. c) In vitro studies: i) Images of polyacrylamide/gelatin (PG) hydrogel vocal folds from stroboscopy showing the opening region of the vocal cold during air flow from the vent. ii) In vitro studies using neural stem cells (NSCs) with live/dead staining in chitosan (CS) and chitosan/hyaluronan (CH) hydrogels after 4 and 72 h of culture. Live and dead cells are indicated with green and red fluorescence, respectively. Few dead cells were observed in the hydrogels after 4 h. d) In vivo studies showing the efficacy of the CH hydrogel in alleviating brain atrophy and neurological deficits in a rat model. Typical weighted images of longitudinal brains from intracerebral hemorrhage (ICH) rats injected with saline or CH hydrogel for two and 14 days. The arrows (↑) represent the brain tissue in the emerging cavity, the arrowheads (^∧^) represent the injected hydrogel, and the star (*) represents liquefied brain tissue. In the rats injected with saline, the remaining brain tissue was found in the emerging cavity after two days, whereas the injured brain tissue remained in the emerging cavity for the CH hydrogel even after 14 days. a, iv) Reproduced with permission.^[^ ^18^ ^]^ Copyright 2023, Nature. a, v) Reproduced with permission.^[^ ^102^ ^]^ Copyright, Elsevier. c, i) Reproduced with permission.^[^ ^28^ ^]^ Copyright 2018, ACS. c, ii and d) Reproduced with permission.^[^ ^116^ ^]^ Copyright 2020, ACS.

Self‐healing can be achieved by using either intrinsic or extrinsic strategies.^[^ ^84^, ^85^ ^]^ In the former, healing occurs because of properties inherent to the material(s) used for the fabrication of the implant.^[^ ^86^ ^]^ In the latter, healing is achieved via mechanisms and materials included in the implant, such as mending materials/agents whose release is triggered by damage to the implant^[^ ^18^, ^19^, ^87^ ^]^ (**Figure** **2**). In the intrinsic approach (Figure 2a(i)), self‐healing can be achieved via chemical and physical mechanisms or a combination of both. Examples of internal self‐healing mechanisms include those based on dynamic covalent (acylhydrazone bonds,^[^ ^88^ ^]^ imine bonds,^[^ ^89^ ^]^ disulfide bonds,^[^ ^90^ ^]^ metal‒organic frameworks, and Diels‐Alder reactions^[^ ^91^ ^]^) or noncovalent (such as hydrogen bonds,^[^ ^92^ ^]^ hydrophobic, guest‒host interactions, and electrostatic interactions^[^ ^93^ ^]^) chemistries or functionalized surface modifications in implants that enhance self‐healing features. Materials utilized to develop self‐healing implants of this category use crosslinking mechanisms, which provide easy fabrication and activate self‐healing processes under mild conditions.^[^ ^94^, ^95^ ^]^ Other approaches involve in situ electrostatic interactions between the positive charge of chitosan and the negative charge of chondroitin sulfate in chitosan and chondroitin sulfate scaffolds.^[^ ^96^ ^]^

In the extrinsic approach (Figure 2a(ii)), external stimuli such as changes in light, temperature, or humidity lead to the release/activation of a healing agent (included as a filler, withincapsules, or in vascular networks),^[^ ^97^, ^98^ ^]^ which initiates the repair/healing process. In one type of external approach, a vascular self‐healing system is used. This system incorporates healing agents within capillaries (e.g., hollow tubes, channels, or nanofibers), which are interconnected in a network‐like structure.^[^ ^99^ ^]^ When such a vascular system is broken, healing agents are released into the system to initiate the self‐healing process. However, these networks can be refilled from external sources or from existing connecting vascular networks.^[^ ^100^ ^]^ Another system is capsule‐based (Figure 2a(ii)), and it consists of micro‐ or nanocapsules that are composed of a polymer matrix. It is based on a polymerization process, which is triggered by mechanical damage to the capsule, after which the release of the encapsulated initiator and reactants takes place. Healing occurs when the healing agents fill the cracks and polymerize.^[^ ^101^ ^]^

In terms of external mechanisms, material‐producing microorganisms have also been investigated.^[^ ^18^, ^19^ ^]^ For example, the use of fungi in 3D‐printed structures to produce self‐healing materials achieved by the growth of mycelia has been demonstrated (Figure 2a(iii,iv)).^[^ ^19^ ^]^ The growth of hyphae across damaged sites allows the structure to self‐heal cracks. Self‐healing can also be achieved by using *Gluconacetobacter xylinus* bacteria‐laden inks, which are derived from cellulose networks (Figure 2a(v)).^[^ ^102^ ^]^ Self‐healing biomaterials may also be made by employing stem cells as building blocks, although such an idea is not practical for typical engineering applications.^[^ ^82^ ^]^

##### Studies—Concept and In Silico Studies

The design of self‐healing methods requires careful consideration of their biocompatibility, mechanical properties, structure, and healing mechanism.^[^ ^103^ ^]^ In an in vitro fatigue simulation study, the effects of cyclic loading on resin‐based composites with acrylic healing liquids containing microcapsule dental composites were investigated, revealing that these composites have increased fatigue resistance.^[^ ^104^ ^]^

##### Studies—Material Studies

In one approach, the effects of coating implants with a self‐healing layer on their behavior and durability were investigated^[^ ^105^, ^106^, ^107^, ^108^, ^109^ ^]^ (**Table** **1**). For example, self‐healing coatings were achieved by embedding polyphenols on the surface of Mg implants.^[^ ^110^ ^]^ When cracks formed in the coating, the polyphenols were released and chelate with the Mg ions to fill the defects (Figure 2c(i)). In addition, the self‐healing matrix of the implant material was investigated.^[^ ^111^ ^]^ This is the first report detailing the development of self‐healing synthetic materials for biomedical applications that were published in 1998 and discussed a new polymer biomaterial containing covalently bound iodine for making a self‐healing drug reservoir.^[^ ^111^ ^]^ Although several studies^[^ ^112^, ^113^, ^114^ ^]^ have discussed the self‐repair ability of materials before this, these studies were not specifically prepared for medical applications. Later, a study published in 1996 revealed that cracks in a matrix material can be repaired via the controlled release of repair agents.^[^ ^113^ ^]^ Although not intended for medical application, a novel polymer composite with a self‐healing ability that can recover its initial toughness by 75% was developed.^[^ ^114^ ^]^ In medical applications, a self‐healing polyacrylamide/gelatin‐based hydrogel was investigated for its use as an artificial vocal fold implant.^[^ ^28^ ^]^ Ex vivo experiments demonstrated successful restoration of the vocal fold (Figure 2c(ii)). For the mechanical evaluation of self‐healing materials, thixotropic studies at different strains and recovery of the self‐healing ability of in situ‐formed scaffolds revealed high viscosity (404.000 mPa s at 0 s and 0.25% strain), indicating a crosslinked structure.^[^ ^96^ ^]^ The viscosity decreased drastically at 1000% strain for 10 s. However, a rapid increase of >90% recovery was observed in the early stage, which indicated quick restoration of the crosslinked structure in the scaffold.

**Table 1:** Summary of self‐healing materials used in the form of coatings and hydrogels, including material type, test/study type, and method(s), application, and main outcome.

Self‐healing implant/material	Other materials	Application	Test/study type and method(s),	Main outcome	Refs.
A. Self‐healing coatings	Coating layer				
Mg‐1Ca implant	Silk fibroin	Coating of Mg alloy‐based bone implants	Scanning vibrating electrode technique (SVET), and electrochemical impedance spectroscopy (EIS) to investigate self‐healing properties.	Fabrication of self‐healing, pH‐sensitive anti‐corrosive silk fibroin, illustrating the preferable biocompatibility, self‐healing properties, and osteogenic activity of the coating.	[108]
Mg‐1Ca implant	Composite coating (silk fibroin‐based)	Self‐healing coatings with corrosion resistance on Mg alloys for bone implant	In vitro corrosion behavior study. self‐healing property study using energy‐dispersive spectrometer (EDS)	This proposed composite coating provides self‐healing properties, and it also has good biodegradation and biocompatibility properties. This study not only presents a significant contribution in enhancing the corrosion resistance of magnesium alloys but also gives novel perspectives on the surface modification of biomedical magnesium alloys	[145]
Mg‐1Ca implant	Functionalized Silk fibroin (silk) based coating consisting of halloysite (HNT)/phytic acid (PA)	Self‐healing biocompatible corrosion resistance coatings for Mg‐based implants	In vitro corrosion behavior analysis by Hank's solution. Self‐healing properties investigated bi EIS after Hank's solution. In Vitro analysis of Biocompatibility and Osteogenic properties The degradation pattern and self‐healing mechanisms of Silk‐HN/PA coating were studied in vivo for bone implant of rat femora	Investigation by histological analysis shows the excellent osseointegration of coated layer on the Mg alloy	[146]
Mg alloy Implant	Polysiloxane modified Composite coating	Anticorrosion and antibacterial coating for bone implant	Cross‐cut immersion tests were conducted to investigate self‐healing properties. Antibacterial behavior was examined using the plate‐counting method	The coating enhances corrosion resistance and possesses antimicrobial performances. The results show that this coating has self‐healing functionality of polysiloxane, which also indicates the potential to advance the use of anticorrosion and antibacterial coatings in the area of Mg alloy implants	[147]
MAO‐coated magnesium alloy AZ31	Mg–Al layered double hydroxide coatings	Orthopedic bone implant materials	The corrosion resistance of the Mg alloy was investigated by the EIS method. In vitro cell culture testing was used to investigate the biocompatibility	In vitro, investigation shows the acceptable cytocompatibility of composite coating on the surface modification magnesium alloy by MAO technique. The MAO‐LDH group has higher cell viability than others, showing better biocompatibility.	[148]
ZE21B Mg alloys stents	Poly (thioctic acid) coatings	ZE21B cardiovascular stents	Corrosion resistance was evaluated via the EIS method. SEM was used to examine self‐healing properties after artificial scratch	Results show that the proposed coating layer, due to self‐healing properties, provides good corrosion protection for ZE21B. Moreover. This coating layer exhibits superior antibacterial/oxidation properties. ZE21B cardiovascular stents modified by the hybrid coating can be employed for arterial stenosis operations	[149]
Mg alloy bone implants	duplex coating of plasma electrolytic oxidization/polydopamine	Dental root implant	Self‐healing of corrosion resistance was investigated by using canning electrochemical microscopy (SECM) and EIS methods	Results show that both SECM and EIS methods are reliable in investigating the mechanism and timing of self‐healing of the protective coating layer. Additionally, the coating layer has excellent corrosion resistance, self‐healing capability, and biocompatibility This coating layer can be a potential candidate for titanium dental root implants	[150]
Mg alloy orthopedic implants	Stimuli‐responsive nanocomposite coating	Mg alloy implants for orthopedic application	The corrosion resistance was evaluated using EIS, employing ZSimpWin software. Confocal laser scanning microscopy (CLSM) was used to examine self‐healing properties. Mouse bone marrow mesenchymal stem cells were considered to investigate wound healing, angiogenesis assays, and biocompatibility in vitro. Eighteen New Zealand white rabbits were considered to investigate in vivo osseointegration.	Nanocomposite coating possesses multiple functions, including bacteria killing, osteogenesis, and angiogenesis. In vitro, results show that better cytocompatibility, osteogenesis, and angiogenesis are observed under near‐infrared. Results reveal that the Stimuli‐responsive nanocomposite coating effectively promotes in vivo osseointegration	[151]
AZ31 Mg alloy	Electrical‐responsive biocompatible coatings	a bioimplant material field such as cardiovascular stent	EIS of scratched coating was employed to investigate the self‐healing properties. The corrosion resistance of coatings was investigated using the EIS test and hydrogen evolution of unscratched coatings	Proposed coatings owning outstanding corrosion resistance, self‐healing, and biocompatibility can be a potential candidate in the field of bioimplants	[105]
B. Self‐healing hydrogels					
Dibenzaldehyde‐terminated ethylene glycol hydrogels (PEGDA)	*N*‐carboxyethyl chitosan (CEC)	Drug delivery for hepatocellular carcinoma therapy	Macroscopic self‐healing test and rheological recovery test.	Injectable self‐healing hydrogel with pH‐responsiveness, self‐healing ability, and good cytocompatibility In vitro observation shows Dox release, which is beneficial for tumor therapy In vivo study shows rapid hydrogel formation after injection of CEC/PEGDA that can be used for cancer treatment	[133]
single component zwitterionic hydrogel	‐	Biomedical applications	Tensile tests were carried out to examine the mechanical properties and effectiveness of self‐healing. In vitro examination of the fusion and biocompatibility of zwitterionic hydrogels using commercial LIVE/DEAD assay kit	Hydrogel has excellent thixotropic property. Able to repair damage independent of time subjected to physiological conditions. Self‐healing properties are observed without the need for any external stimulus In vitro study shows that it is important to highlight that the process of zwitterionic fusion did not result in a substantial decrease in cell viability	[152]
Injectable micelle/hydrogel composites	‐	Skin wound healing	In vitro study to examine drug release capability Female Kunming mice were used to investigate the wound healing capability‐ an in vivo study Antibacterial activity was examined using *Escherichia coli* and *Staphylococcus aureus* methods	Appropriate stretchable and similar modulus with human skin, decent adhesiveness. The hydrogels show good self‐healing properties and biocompatibility The hydrogel with curcumin accelerated the wound healing rate LIVE/DEAD Viability/Cytotoxicity Kit assay shows good cytocompatibility	[135]
Polymer network (IPN) hydrogel	Developed based on polyacrylamide and gelatin	Voice Recovery	A compression test was conducted to study hydrogels' mechanical and rheological properties *Ex vivo* study investigates the function of hydrogel for artificial vocal fold tissue (porcine skin)	Considerable resistance to the dehydrated environment. Self‐healing and safe biocompatibility properties Recovery of vocal tissues was observed after the implantation of the hydrogel	[28]
Injectable thermosensitive magnetic hydrogel	Based on ethylene glycol	Drug delivery for chemotherapy in cancer treatment	In vivo results prove the proposed Hydrogel's antitumor efficacy (Female BALB/c mice). In vitro study on L‐929 cells	Excellent biocompatibility, exceptional self‐healing, injectable, good magnetic responsive, and heat‐inducing properties. In vivo results prove the antitumor efficacy of the proposed Hydrogel. In vitro, results show the biocompatibility and drug release of DOX. Both in vitro and in vivo studies exhibit enhanced synergistic antitumor activity	[134]
Hydrogel based on sodium hyaluronate oxide (SAO) and polyaniline‐grafted gelatin.	Neural stem cells and donepezil	Spinal cord injury treatment	In vivo study on Female Sprague Dawley rats.	Results show self‐healing, biocompatibility, electroactive The use of injectable hydrogels together with stem cells/drugs has shown advancements in the field of spinal cord injury (SCI) healing. This approach exhibits promise in addressing the limitations associated with conventional drug‐based treatments and stem cell therapies	[139]
Hydrogel based on the dopamine/β‐cyclodextrin‐modified hyaluronic acid (HA‐CD‐DA) and amantadine modified carboxymethyl chitosan	Loaded by dexamethasone (DEX)	Ulcerative colitis therapy	Inverted optical microscope analysis for self‐healing properties evaluation. In vitro (immortalized human epithelial cell line Caco‐2, immortalized macrophage line RAW264.7and normal human colon mucosal epithelial cell line NCM‐460) degradation and Biocompatibility analysis of hydrogels In vivo studies were done on mice (7‐week‐old) degradation, anti‐inflammatory efficiency, and Biocompatibility analysis hydrogels	Results show self‐healing, biocompatibility, and anti‐inflammation hydrogels. The findings of this study demonstrate that the developed self‐healable hydrogel effectively improved the symptoms of ulcerative colitis in mice models produced by dextran sulfate sodium (DSS) with a single dose of hydrogel. Moreover, developed hydrogel shows remarkable potential as a treatment strategy for UC therapy in clinical environments	[153]
O‑carboxymethyl chitosan hydrogel	Tannic acid modified gold nanocrosslinker	Treating Parkinson's disease	The SEM image examination of self‐repairing properties In vitro analysis assay of antioxidative and anti‐inflammatory In vivo analysis effectiveness of hydrogel on a rat model of Parkinson's disease	Results prove Anti‐inflammatory, antioxidative capabilities, injectable, self‐healing Results show that bioactive hydrogel facilitates motor function restoration and mitigates histological neurodegeneration in rats with Parkinson's disease. Notably, the effectiveness of the O‑carboxymethyl chitosan hydrogel implant was comparable to that of the drug‐loaded hydrogel. The results of this study provide evidence that the bioactive and conductive O‑carboxymethyl chitosan hydrogel has potential as a biomaterial implant for neuroprotection and Parkinson's disease treatment beyond its conventional role as a carrier for cells or drugs	[154]

##### Studies—In Vitro Studies

In vitro studies of the self‐healing properties of in situ‐formed scaffolds made of chitosan/chondroitin sulfate revealed significant increases in the proliferation and migration of human keratinocytes and human dermal fibroblasts grown on the scaffolds, indicating the biocompatibility of the material and its mechanical support.^[^ ^96^ ^]^ In another study, enhanced healing of in vitro wounds (human dermal fibroblasts and keratinocyte cell lines) that were treated in situ to form self‐healing polyelectrolyte complexation scaffolds was demonstrated. Compared with that in cells grown on chitosan (CH) or chondroitin sulfate (CS), the expression of proliferating cell nuclear antigen (PCNA) in these cells was significantly greater.^[^ ^115^ ^]^ The use of self‐healing chitosan‐hyaluronan (HA) hydrogels to encapsulate neural stem cells (NSCs) has demonstrated a conducive microenvironment for neural regeneration.^[^ ^116^ ^]^ With increasing HA content in the scaffolds, NSCs migrated more and spread across the scaffold.

##### Studies—In Vivo Studies

In vivo experiments have investigated the self‐healing properties, biocompatibility,^[^ ^85^, ^117^ ^]^ and efficacy^[^ ^116^, ^117^, ^118^, ^119^ ^]^ of implanted biomaterials. The tested biomaterials include polysiloxane,^[^ ^85^ ^]^ polyurethane,^[^ ^117^, ^120^, ^121^ ^]^ polyethylene glycol,^[^ ^118^ ^]^ chitosan/chondroitin sulfate,^[^ ^96^ ^]^ and chitosan–hyaluronan,^[^ ^116^ ^]^ which are implanted subcutaneously in mice^[^ ^85^, ^117^ ^]^ and rats^[^ ^117^ ^]^ or are applied for treating certain conditions, such as aortic aneurysm or brain injury.^[^ ^118^ ^]^

For example, polysiloxane sheets cross‐linked with Diels–Alder bonds were found to have excellent self‐healing properties and were nontoxic when they were implanted subcutaneously in mice.^[^ ^85^ ^]^ The biocompatibility of self‐healing polyurethane sheets (healing is based on hybrid dynamic oxime‒urethane covalent bonds and hydrogen bonds) was demonstrated after their subcutaneous implantation in mice and rats.^[^ ^117^ ^]^ Their efficacy in treating aortic aneurysms (mice), nerve coaptation (rats), and bone immobilization (rats) has also been shown. Self‐healing polyurethane implants have been shown to be nontoxic^[^ ^120^ ^]^ and useful for use in minimally invasive surgery. A polyurethane film was thus applied on the surface of the heart in the beagles.

Self‐healing scaffolds consisting of polycaprolactone (PCL)‐gelatin methacryloyl (GelMA) nanofibers containing dibenzaldehyde‐terminated polyethylene glycol (GC‐DFPEG) hydrogels loaded with bone marrow‐derived stem cells (BMSCs) were implanted into ischemic areas of the brain (after occlusion of the middle cerebral artery) in rats. BMSCs migrated from the scaffolds into the injured area, leading to a significant reduction in the infarct area.^[^ ^118^ ^]^ Compared with acellular scaffolds, MSC‐laden scaffolds led to decreased brain inflammation and increased neurogenesis and angiogenesis. Rats that received cell‐containing self‐healing scaffolds presented significant reductions in infarct volume, extent of brain edema, and neurological deficits.

In vivo studies in mice demonstrated the efficiency of self‐healing polyurethane elastomer sheets in treating aortic aneurysms, and nerve coaptation and bone immobilization were shown.^[^ ^119^ ^]^ In vivo studies of rat skin excisional wounds have shown that treatment with a self‐healing chitosan/chondroitin sulfate scaffold is associated with faster wound closure, increased collagen content, and alpha smooth muscle actin (α‐SMA) and beta 1 (β_1_)‐integrin expression.^[^ ^96^ ^]^ The wound width was ≈89.5% on day 10, and histological evaluation of the wounds performed at 7 and 14 days revealed significantly improved reepithelization.

The efficacy of the self‐healing chitosan‐hyaluronan hydrogel injected into the zebrafish brain following traumatic injury (TBI, cavity created in the brain) led to better functional recovery (as indicated by swimming activity) than that of the chitosan or saline injection alone into the brain cavity.^[^ ^116^ ^]^ The efficacy of the hydrogel was further evaluated in a rat model. Chitosan‐hyaluronan hydrogel injection led to the maintenance of brain tissue in the injury‐associated cavity at 14 days, whereas saline injection led to the liquefaction of brain tissue at this time. Moreover, the rats treated with the self‐healing hydrogel recovered >70% of their behavioral function within 14 days, which was significantly greater than that observed after saline injection (Figure 2d).

##### Studies—Clinical Studies

To date, no clinical reports on self‐healing implants have been published. However, there is a phase III clinical trial (beginning in June 2022, ending in December 2022) on the use of self‐healing hydrogels for the delivery of recombinant human epidermal growth factor for the treatment of oral mucositis associated with cancer therapy.^[^ ^122^ ^]^ No publications reporting results have been identified.

##### Summary

Different self‐healing strategies, such as nonliving (internal or external mechanisms) and living (fungi, bacteria, or other cell type‐based) methods, have been explored. Material‐based systems are used as the matrix or coatings of the implants. Despite the positive trajectory of self‐healing materials, research has focused mainly on hydrogels, while the development of self‐healing autonomous implants is still in its way. Self‐healing materials have been studied in chemical systems.^[^ ^48^, ^57^, ^94^, ^123^, ^124^, ^125^, ^126^, ^127^, ^128^, ^129^, ^130^, ^131^ ^]^ Further in vitro^[^ ^117^, ^118^ ^]^ and in vivo^[^ ^85^, ^118^, ^119^, ^132^ ^]^ studies were used to examine the biocompatibility, toxicity, robustness, and reproducibility of the implants.^[^ ^132^ ^]^ Potential applications include cancer treatment,^[^ ^133^, ^134^ ^]^ wound healing,^[^ ^135^, ^136^ ^]^ treating brain diseases,^[^ ^137^, ^138^ ^]^ and treating spinal cord injury.^[^ ^139^, ^140^ ^]^ In addition, they can be used to develop stretchable self‐healing skin meshes,^[^ ^141^ ^]^ soft robotics,^[^ ^142^ ^]^ and wearable sensing devices.^[^ ^143^ ^]^ Because of concerns related to toxicity, robustness, and reproducibility, which have not yet been addressed,^[^ ^125^, ^137^, ^144^ ^]^ no clinical applications have been pursued. Self‐healing biomaterials enable the execution of stitchless surgical procedures, allowing procedures to be carried out in narrow surgical fields, such as in endoscopic surgery or intracranial operations. This makes them attractive tools that will impact future surgery.

#### 2.2.2 Self‐Regeneration

A self‐regenerating implant is defined as an implant that has the ability to regenerate after it decays in part or in whole. The purpose of self‐regeneration is to enable continued function of the implant, such as sensing,^[^ ^155^, ^156^, ^157^, ^158^ ^]^ actuation,^[^ ^159^ ^]^ or other functions.^[^ ^160^ ^]^ Research in the area of self‐regeneration of implants is relatively limited.

##### Mechanisms

**Figure 3:**
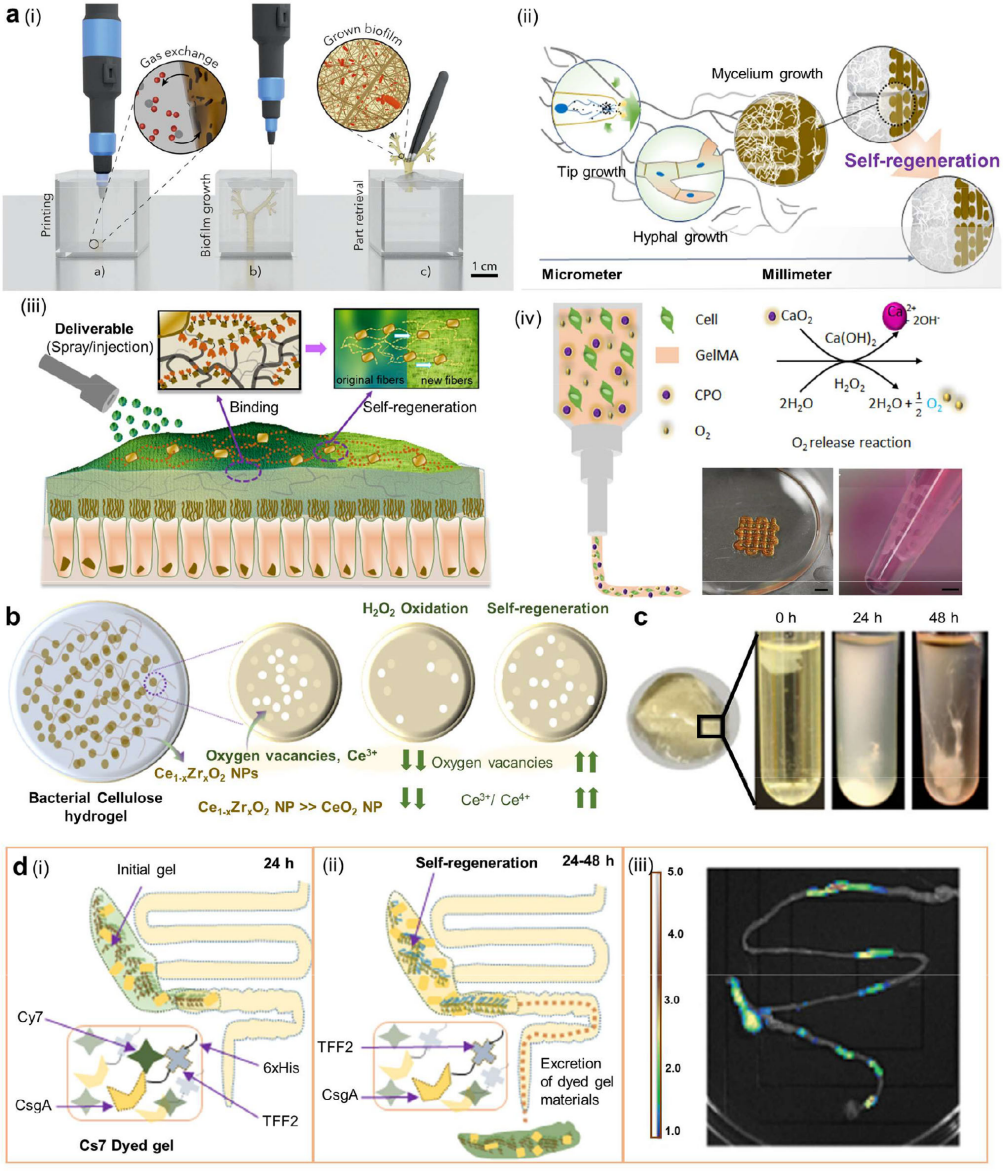
Self‐regeneration of implants can be achieved through a) mechanisms: i) a biomaterial‐based approach, which includes a cellulose‐based living structure; ii) a fungus‐based approach, which involves self‐regeneration via mycelial cell growth, which results in the formation of a hyphal network and grid‐shaped structures; and iii) a bacteria‐based approach,^[^ ^162^ ^]^ which involves keeping the bacteria alive in the printed object. The engineered living materials developed complex geometries capable of self‐regeneration of their cellulose fiber network following damage. The aerobic bacteria *Gluconacetobacter xylinus* were embedded in aqueous ink to produce cellulose fiber networks after printing into the desired geometry. iv) Tissue engineering was based on O_2_‐generating biomaterials, where cells and O_2_ sources containing bioinks were combined to fabricate 3D‐printed constructs for regenerative therapy. b) Material studies: bacterial cellulose hydrogels containing Ce_1‐x_Zr_x_O_2_ NPs with self‐regenerating antioxidant properties whose efficiency depends on the equilibrium population of Ce^3+^ and Ce^4+^. c) In vitro studies: i) live hydrogels harvested in bacterial growth medium. The dry mass of the live gels was measured after 24 and 48 h in the presence of various concentrations of SDS. d) In vivo studies: i) Illustration of the self‐regeneration process of live gels in the GI tract of mice.^[^ ^162^ ^]^ Live gel that was administered orally to mice after 24, 48, and 72 h was tracked. Cy5 fluorescence was measured in GI tracts harvested 72 h after live gel administration. A fluorescent dye was conjugated to Ni‐NTA, and its concentration in harvested GI tracts was observed after 72 hours. The live gel was composed of TFF2 protein and 6x‐His tags at the C‐terminus. The live gel retained the dye and was observed in the cecum and proximal and medial colon of the mice, whereas the original curli fibers of the live gel were excreted completely by the dye. a,i) Reproduced with permission.^[^ ^18^ ^]^ Copyright 2023, Nature. ii) Reproduced with permission.^[^ ^162^ ^]^ Copyright 2019, Wiley‐VCH; b,c) Reproduced with permission.^[^ ^162^ ^]^ Copyright 2019, Wiley‐VCH.

Approaches include biomaterial‐based^[^ ^102^ ^]^ and living cell‐based approaches to achieve self‐regeneration in an implant.^[^ ^18^, ^19^ ^]^ Biomaterial‐based systems include mineralized hydrogels and mineral‒polymer composites (**Figure** **3**a(i)). Cell‐based systems include microorganism‐assisted approaches, such as the use of fungi (Figure 3a(ii))^[^ ^161^ ^]^ or bacteria (Figure 3a(iii)),^[^ ^102^ ^]^ to synthesize a biomaterial^[^ ^162^ ^]^ or form structural elements to regenerate the construct. For example, one can use *Escherichia coli* to produce nanofibrous structures,^[^ ^102^, ^162^, ^163^, ^164^ ^]^ or fungi to extend hyphae, which in turn can form 3D network structures and help regenerate constructs (Figure 3a(ii)).^[^ ^18^ ^]^

##### Studies—Concept

Polyacrylamide hydrogels can regenerate swollen surfaces after abrasive wear to maintain a low friction surface ability.^[^ ^165^ ^]^ This ability of polyacrylamide hydrogels can be exploited for developing biomedical devices that need to maintain their surface at low friction.

##### Studies—Material Level—Material‐Based

In one example, self‐regeneration in an ionotronics hydrogel was achieved via MXene and calcium chloride (CaCl_2_), which endow the hydrogel with both ion‐ and electron‐conductive channels.^[^ ^166^ ^]^ Self‐regenerative ability was attained by the incorporation of CaCl_2,_ which has the ability to retain water for a longer duration. The hydrogel retained water for up to 70 days of continuous use, which is essential for the long‐term stability of the hydrogel‐based bionic skin sensor. Furthermore, the hydrogel exhibited frost resistance at ultralow temperatures (─50 °C) and exhibited a sensing ability at lower temperatures.

##### Studies—Material Level—Cell‐Based

To produce 3D structures, a cellulose‐producing bacteria, *Gluconacetobacter xylinus*, was used in bioinks to 3D bioprint a bronchial tree‐like structure (Figure 3).^[^ ^102^ ^]^ Nutrients for bacterial growth are already present in the ink, which enables the formation of cellulose fibers. The oxygen (O_2_) required by microorganisms for metabolism is obtained from air.^[^ ^167^ ^]^ In another study, *Gluconacetobacter xylinus* was embedded in aqueous ink (xanthum gum in culture medium) to produce a cellulose fiber network in a silicone‐based granular gel.^[^ ^168^ ^]^ The bacteria remained alive in the printed constructs, which were capable of self‐regeneration of their cellulose fiber networks after network decay. Furthermore, complex tubular structures were printed, incubated for bacterial growth and cellulose formation, and cut with a blade. When the cut ends were placed in close contact and incubated in culture medium for three days, regeneration was observed. The nutrients in the medium enabled the growth of cellulose fibers, regenerating the fiber network of the damaged hydrogel. The growth of cellulose fibers occurred throughout the structure, which also resulted in a smooth surface and thickening of the original geometry of the fibers. The challenge is what stops these bacteria from producing more, or they will be stopped by filling voids in adjacent structures and close vicinity only.

For example, bacterial cellulose composites were developed by functionalizing cellulose with cerium oxide (CeO_2_) nanoparticles to achieve self‐regeneration properties.^[^ ^169^ ^]^ Furthermore, zirconium was incorporated into the CeO_2_ lattice to increase the O_2_ storage capability of these composites. This was demonstrated via diffuse‐reflectance UV–vis tracking of composite absorbance over two weeks while the composite was immersed in water (H_2_O_2_). The absorption edge was dependent on the oxidation state of cerium. When the composites were immersed in H_2_O_2_, there was a shift in the absorption when Ce^3+^ was converted to Ce^4+^, and the wavelength shifted upon oxidation and subsequently recovered the original absorption edge wavelength, which indicated the regenerated nature of the composite materials. Compared with those within the body, complete regeneration was observed after two weeks, despite the greater concentration (Figure 3b). In a recent study,^[^ ^162^ ^]^ self‐regeneration hydrogels were fabricated using *E. coli* as the cellular chassis and mucoadhesive protein nanofibers as the extracellular matrix (ECM). The bacterial hydrogel was harvested from broth cultures via filtration. The hydrogel regenerated itself in situ and grew fourfold in mass over a period of 24 h when optimal conditions were maintained. The hydrogel was programmed to interact with different specific tissues in the gastrointestinal tract (GI) owing to its genetically programmable rheological properties, which enable it to be retained in the GI tract for several days because of continual regeneration, such as the mucins themselves.

In 3D bioprinted constructs that contained Ganoderma Lucidum embedded in hydrogels (agar, κ‐carrageenan, and cellulose‐based thickener), mycelia grew between hydrogel filaments after 20 days of incubation.^[^ ^18^ ^]^ The growth of mycelia between the filaments was strongly influenced by the malt concentration present in the substrate (Figure 3a). Mycelia grew by 0.6–0.7 mm day^−1^ and reached a maximum healing distance of 2.5–3 mm when malt was available at a concentration equal to or greater than 6%. Interestingly, mycelial network formation and metabolic activity can be regained even after a prolonged dormant state of as long as 8 months.^[^ ^19^ ^]^

##### Studies—In Vitro Cell Studies

Cell viability assays using human colonic carcinoma cell lines (Caco‐2 cells) coincubated with self‐regenerating curli fiber hydrogel gels that were obtained from bacterial culture confirmed their lack of cytotoxicity (Figure 3c).^[^ ^162^ ^]^

##### Studies—In Vivo Studies

To investigate the mucoadhesive properties of self‐regenerating living gels, PQN4 bacterial cells entrapped in the curli‐specific gene (CsgA) protein‐trefoil factor family 2 (CsgA‐TFF2) curli fiber network were orally administered to healthy mice, which were monitored by harvesting tissues at different endpoints (Figure 3d).^[^ ^162^ ^]^ The study demonstrated that curli fiber hydrogel was expelled after 24 hours, whereas the embedded bacteria were retained within the GI tract, allowing continuous regeneration of fibers.

##### Studies—Clinical studies

To the best of our knowledge, no clinical studies have been reported.

According to the Food and Drug Administration (FDA), medical implants are defined as devices or tissues that are placed inside or on the surface of the body. Many implants are prosthetics intended to replace missing body parts. Other implants can be used to deliver medication, monitor body functions, or provide support to organs and tissues.^[^ ^170^ ^]^ Devices are regulated differently from tissues, which have more challenging regulatory pathways.^[^ ^171^, ^172^ ^]^ Chen et al. provide a comparative overview of how the U.S., EU, Japan, and China regulate tissue engineering and regenerative medicine (TERM) for clinical use. In the U.S. and EU, regulators follow a risk‐based approach, minimally manipulated, homologously used cell procedures often fall under medical techniques, i.e., less regulated, whereas applications involving more manipulation or combination with biomaterials are treated as medical products, requiring full oversight. Japan offers some flexibility for clinical research with processed stem cells despite the lack of a formal approval pathway.^[^ ^171^ ^]^

##### Summary

Several approaches can be used to achieve self‐regeneration properties in hydrogels,^[^ ^165^, ^173^, ^174^ ^]^ and coatings,^[^ ^175^, ^176^, ^177^, ^178^ ^]^ via the use of biomaterial‐producing bacteria and fungi.^[^ ^161^ ^]^ To date, no studies have used cells other than bacteria to examine their cytocompatibility or efficacy, or animal studies. These studies are essential for demonstrating the safety and value of self‐regenerating biomaterials, taking them forward toward integration into smart materials and enabling the development of autonomous implants in the future.

#### 2.2.3 Self‐Reconfiguration and Transformation

**Figure 4:**
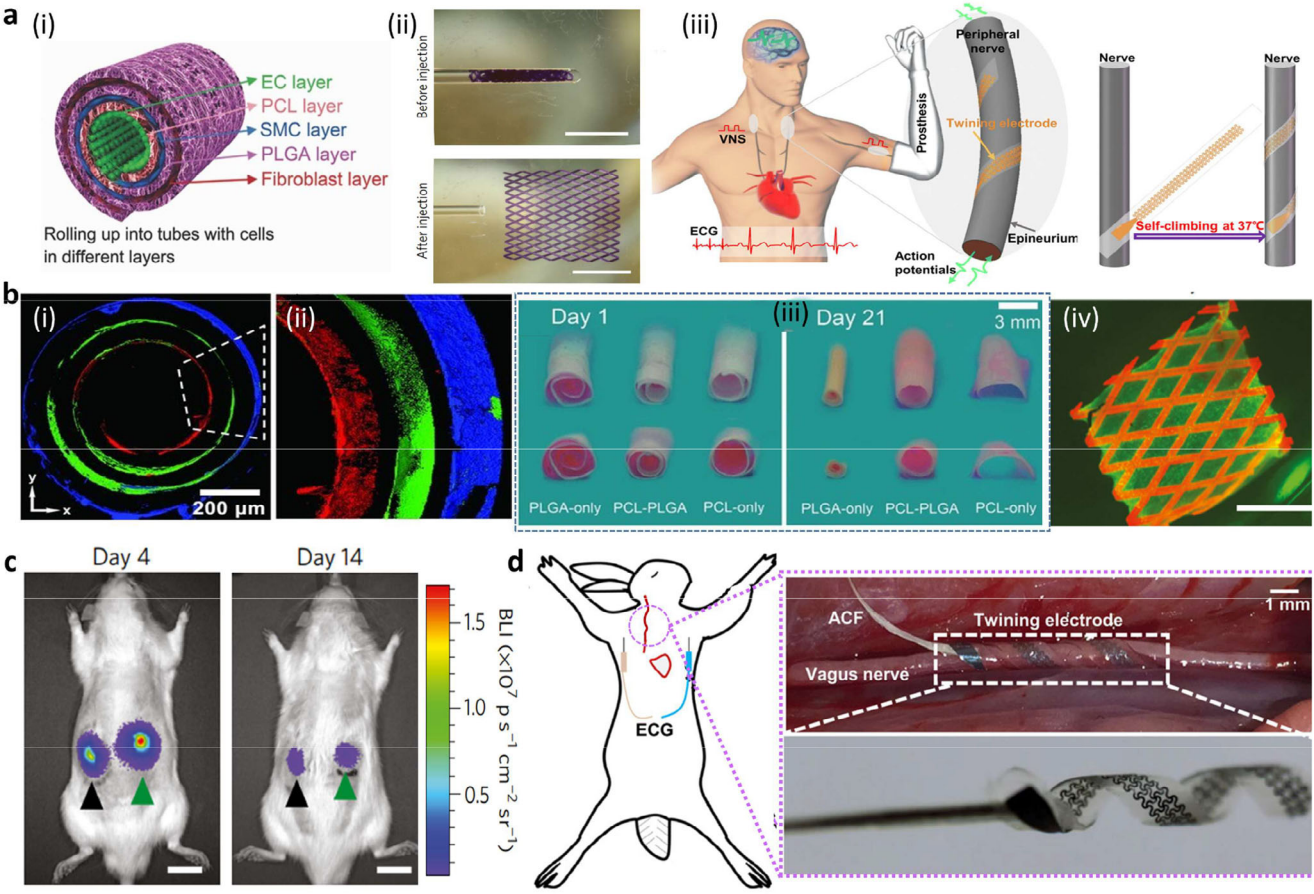
Self‐configurating/self‐forming implants. a) Mechanisms. i) Illustration of stress‐induced rolling membrane (SIRM) technology to fabricate 3D patterns on tubes that contain cells (smooth muscle cells (SMCs), endothelial cells (ECs), and fibroblast layers), a polycaprolactone (PCL) layer, and a poly(lactide‐co‐glycolide) (PLGA) layer in a biomimetic fashion. ii) Shape memory mechanism, showing images of the poly(octamethylene maleate (anhydride) citrate) scaffold before and after injection through a glass capillary. iii) Self‐climbing mechanism: Schematic representation of peripheral nerves (left) and the formation of an electrode–nerve interface, self‐climbing of the electrode from a flattened state driven by body temperature. b) Cell studies: i) Distribution of three types of cells (red: human umbilical vein endothelial cells; green: smooth muscle cells (SMCs); blue: fibroblasts) from inside after rolling SIRM, indicating a similar pattern of blood vessels. ii) Magnification and side view of the tube. iii) Images of cell‐laden PLGA‐tube, PCL‐PLGA, and PCL tubes cultured in Dulbecco's modified Eagle's medium (DMEM) for different durations. PCL‐PLGA maintained its regular shape for 21 days, and the three layers of PCL (one layer) and PLGA (two layers) merged into a single layer. PLGA and PCL tubes underwent significant transformation. The inner and outer diameters of the PLGA tube decreased, whereas the PCL tube gradually swelled, leading to the collapse of the tubular structure in both the PCL and PLGA tubes. iv) Fluorescence image of the POMAC scaffold containing live (green) and dead (red) rat cardiomyocytes after injection. The POMAC scaffold exhibited autofluorescence in the red channels. Live cells filling the scaffold lattice are shown in green. c) In vivo studies: Bioluminescence imaging after subcutaneous implantation of surgically implanted (black arrow) and injected (green arrow) rat cardiac tissues. The cells remained localized following subcutaneous injection, and no significant difference was observed between the two cardiac patches after 14 days of implantation. d) Application studies: Schematic diagram of vagus nerve stimulation (VNS) and implantation of a twining electrode on the vagus nerve in a rabbit model. A twining electrode with an inner diameter of 1 mm was implanted, as it is equal to the diameter of the vagus nerve of a rabbit. a, i) and b, iii) Reproduced with permission.^[^ ^200^ ^]^ Copyright 2017, Wiley‐VCH. B, i, ii) Reproduced with permission. Copyright 2012, Wiley‐VCH. a, ii, iv, c) Reproduced with permission.^[^ ^201^ ^]^ Copyright 2017, Nature. a, iii, d) Reproduced with permission.^[^ ^202^ ^]^ Copyright 2019, Science.

A self‐transforming implant is defined as the ability of an implant to change its physical properties as needed in self‐acting internal force‐driven mode without the need for external intervention. These transformations include morphological/mechanical changes from an initial two‐dimensional (2D) shape to a predesigned 3D shape,^[^ ^179^, ^180^, ^181^, ^182^, ^183^, ^184^, ^185^, ^186^, ^187^, ^188^ ^]^ from a relatively low viscosity to a semisolid material, from a low rigidity and compliance to a high rigidity state,^[^ ^189^ ^]^ or from small parts to predetermined assembly (**Figure** **4**). Recently, considerable interest has been given to self‐forming/self‐reconfigurable 3D implants, which are inspired by naturally occurring blood and lymph vessels. Biodegradable in situ‐forming implants (ISFIs) formed from injectable fluids have advantages, as the procedure is less invasive. They can be successfully used for in situ tissue engineering, cell culture and transportation, and drug delivery.^[^ ^190^, ^191^, ^192^ ^]^ The purpose of having a self‐transforming implant is to mimic the dynamicity of tissues and enable the implant to change form or properties to adapt to changes in its environment or increasing requirements. This includes changes in shape in their rigidity from rigid to soft, to accommodate micromotion or from soft to rigid, to achieve variable‐stiffness actuation. The use of self‐forming/configuring materials also enables minimally invasive implantation and in situ implant formation. These smart implants can successfully adapt to complex tissue surfaces and structures.

Self‐adaptive implants are also important for fighting infections, as they help to avoid conventional methods of infection prevention and their side effects, e.g., the use of implants as surface coatings^[^ ^193^, ^194^ ^]^ or functionalization^[^ ^193^, ^195^ ^]^ strategies. Self‐adaptive implants were therefore introduced to address infection, e.g., of the bone.^[^ ^196^ ^]^ In this case, hydroxyapatite (HAp) was used as a sample material to establish self‐adaptive properties, and ethylenediamine‐functionalized poly(glycidyl methacrylate) (PGED) brushes were grafted from HAp via surface‐initiated radical polymerization. The antibiotic gentamicin sulfate (GS) was subsequently conjugated to the PGED to produce self‐adaptive and sustainable antibacterial HAp implants.

##### Mechanism

Self‐reconfiguration, including changes in shape and stiffness, can be modulated as a result of strain engineering,^[^ ^179^, ^181^ ^]^ a shape memory polymer‐based strategy,^[^ ^180^, ^182^, ^183^, ^184^ ^]^ a temperature‐triggered soft‒rigid phase transition of the phase change metal gallium,^[^ ^197^ ^]^ gelatin swelling,^[^ ^186^, ^188^ ^]^ and stiffness variation via quick mineralization.^[^ ^189^ ^]^ Self‐assembly can be realized by the magnetic force among magnets to form a predetermined macroconfiguration^[^ ^198^ ^]^ (**Table** **2**).

**Table 2:** A summary of the mechanisms and example applications of self‐forming/self‐reconfigurable implants.

No.	Mechanism	How does the system work?	Applications	Comment	Refs.
1	Strain engineering	The release of a prestressed inorganic nanomembrane deposited at low temperatures onto a polymer sacrificial layer occurs after the removal of the sacrificial layer using a solvent. The membrane then rolls‐up into a tube	Two‐dimensional (2D) confined channels for the guidance of cell growth	A generic approach and different materials that have tunable diameters and lengths are used to produce tubular micro/nanostructures	[179]
		Stress‐induced rolling membrane (SIRM) is fabricated by bonding two elastic membranes together and the top membrane is stretched to produce stress. Different cell types are delivered and patterned on a 2D SIRM using microfluidic channels. SRIM is then released to roll up into a tube	Different cell types are used for the fabrication of multilayered tubular structures, such as blood vessels	A strategy to produce tubular structures that have layered walls composed of different cell types. This allows precise control of cell location and orientation. The strategy enables 3D micro/nanofabrication by using initial patterning in 2D form with subsequent transformation into a 3D form	[181]
2	Shape memory polymer	An elastic shape memory scaffold is developed by using a microfabricated lattice, which allows the engineered tissue to collapse during injection, and regain its original shape when placed at the desired location. Cell viability and tissue function are preserved during the process	Engineered organ‐specific tissue patches for the liver, aorta and epicardium	A general method to fabricate flexible tissue patches for in vivo minimally invasive delivery	[182]
		The shape‐morphing scaffolds consist of a shaping layer composed of SMPs and a function layer. The shaping layer effectively achieved programmed deformation of the endothelial cell‐laden constructs from temporary planar shapes to closed‐hoop tubular shapes at the temperature (*T_trans_*) of 37 ^o^C	Small‐diameter vascular grafts with improved endothelialization	By combining shape memory polymers (SMPs) and tissue engineering scaffolds, one can have a versatile method for achieving programmed deformation of cell‐laden constructs into on‐demand preplanned 3D structures. This will enable the engineering of complex cell‐scaffold constructs that mimic native tissues and organs	[180]
		A three‐dimensional (3D) twining electrode fabricated by using integrating stretchable mesh serpentine wires onto a flexible shape memory substrate. Its shape memory leads to recovery of its shape at the body temperature. Temporarily flat stiff twining electrode will self‐climb onto nerves at the temperature of 37 °C to form 3D flexible neural interfaces that exert minimal constraint on the nerves	Electrodes for peripheral nerve stimulation and recording	Proposed twining electrodes can reduce the risk of nerve injury that may occur due to mechanical and/or geometrical mismatches	[184]
		Fabrication of organic thin‐film transistors on SMPs allows for the fabrication of electronics on a rigid substrate which can soften subsequently and change shape after heating above the *T_trans_* of the SMPs (here 37 °C in the body)	Implantable electronics with mechanical adaptability	These mechanically adaptable electronics have the potential to enable new means of creating intimate interfaces between body tissues and bioelectronics	[183]
3	Phase change metal	Temperature‐triggered transition of soft‐rigid phase of mechanically tunable electronics fabricated with gallium	Deep brain neural probe that remains rigid at the temperature of operating room and softens after deep brain injection	This approach has reduced lesion size and inflammatory glial responses, compared to tungsten needles having the same dimensions, making it more amenable to deep tissue embedding	[197]
4	In situ forming implants based on solvent induced phase inversion	Water‐insoluble biodegradable polymers dissolved in organic and water‐miscible solvents. After injection, the organic solvent dissipates out and the water ingresses, resulting in the solidification of the polymers via phase separation	Tissue repair scaffolds, cell encapsulation, and bioengineering and drug delivery	These systems have several advantages that the need for critical temperature, presence of ions, and change in pH is not required	[233]
5	In situ forming implants based on photopolymerized crosslinking	Crosslinking of monomers with a minimum of two free radicals or cross‐linkable polymers and a photoinitiator under light irritation	Tissue repair scaffolds, cell encapsulation, and bioengineering and drug delivery	These systems have the advantages of injectability facilitating minimal invasive surgery, while the limited penetration depth of light in tissue hinders their broad use	[234]
6	Gelatin swelling	Microscale hollow tubules formed by curling planar hydrogel materials driven by the difference in con‐ tractility and swelling ratio between its upper and lower layers	Biomimetic microvessels	A facile method to fabricate biomimetic microvessel scaffold with high accuracy, controllability, and handleability	[186]
		A hydrogel‐based actuated electrode array was designed using a thin‐film electrode carrier and hydrogel‐actuated shaft, consisting of silicon rubber and dispersed polyacrylamide hydrogel particles. These particles swell after implantation and induce shaft elongation, which leads to curling of electrode array across its axis	Cochlear implants	This approach is hoped to achieve both easy implantation and close contact with the nerve cells	[188]
7	Stiffness varying via quick mineralization	Electromechanically active material is combined with compliant alginate gels. Cell‐derived plasma membrane nanofragments (PMNFs) that can mineralize within 2 days are used to functionalize the gels, which promote the mineralization and stiffness in the gels that helps to design actuators of variable stiffness	Tools for bone repair or bone tissue engineering	PMNF‐based biohybrid materials could simplify the process of building soft‐robotic devices with multiple features (such as phase‐changing capabilities, different morphology) that could be used in tissue engineering applications including robot assisted surgical procedures and morphing bioadhesives	[189]
8	Magnetic force	Magnets self‐assemble into a predetermined macro configuration	Device for intestinal bypass creation	The technology enables the development of self‐assembling magnets that can be used during endoscopic surgeries (for example dual‐path bypass)	[198]

Self‐reconfigurable membranes provide a feasible strategy to fabricate such tubular structures of different diameters.^[^ ^179^, ^180^, ^181^, ^186^ ^]^ Different cell types are used as different layers of tube walls by first patterning different cells on a 2D surface, which can undergo changes in shape, and then deforming the 2D surface into a 3D structure.^[^ ^179^, ^180^, ^181^ ^]^ For example, the technique of stress‐induced rolling membrane (SIRM) was adopted to produce tubular structures that have walls made of layers of different cell types that mimic blood vessels.^[^ ^199^ ^]^ Tubular structures such as the trachea, blood vessels, and lymph vessels have specific 3D shapes and multiple cells at specific locations. The developed tubular structures had different layers containing cells aligned either longitudinally or circumferentially around the tube, similar to the natural arrangement found in native tissues. Tubes with controllable sizes were produced, and the orientation of the cells inside the tubes was regulated by topographical contact guidance. By rolling simple patterns, 2D membranes were transformed into complex 3D tubes. This method is also suitable for the fabrication of self‐adjustable scaffolds that require the maintenance of a defined shape for longer durations (Figure 4a(i)).^[^ ^200^ ^]^ The use of a shape memory scaffold is another example that allows the development of engineered cardiac patches, which can be injected into the body via a minimally invasive approach. The injected scaffold collapsed during injection and regained its original shape following delivery to the target location (Figure 4a(ii)).^[^ ^201^ ^]^ A biocompatible twining electrode with the ability to self‐climb on the basis of body temperature to form 3D neural interfaces is another example (Figure 4a(iii)). These electrodes can reduce the risk of nerve injury, which may be caused by mechanical mismatch,^[^ ^202^ ^]^ because of their self‐reconfiguration. When peripheral nerves undergo deformation, such as bending, swelling, or stretching, the twining electrodes deform, and thus, the risk of nerve damage by the electrodes can be avoided.

Stimuli‐responsive materials enabled the development of 4D printing to produce 4D implants, which undergo changes in shape or function in response to certain triggers that can be local, such as changes in pH or temperature, or external, such as magnetic, electrical, or acoustic stimulation.^[^ ^203^, ^204^, ^205^ ^]^ Once the trigger is removed, these implants autonomously return to their original shape. This behavior has enabled the creation of novel implants such as recoverable sandwich panels and energy‐absorbing meta‐structures, and most recent medical implants combine high impact tolerance with fast shape recovery, illustrating both the design versatility and practical promise of 4D‐printed shape‐memory design.^[^ ^206^, ^207^ ^]^

The principal classes of 4D‐printable biomaterials include shape‐memory polymers (SMPs),^[^ ^206^, ^207^ ^]^ hydrogels, liquid crystalline materials (LCDs),^[^ ^208^, ^209^, ^210^, ^211^, ^212^, ^213^, ^214^ ^]^ and shape‐memory alloys (SMAs).^[^ ^215^ ^]^ For example, temperature‐responsive SMPs are used in 4D printing because of their desirable glass transition temperatures (*T*_g_), which range from −70 to 150 °C.^[^ ^216^ ^]^ To tailor SMPs for biomedical use, where activation near body temperature is needed, their *T*_g_ can be precisely adjusted either by modifying the polymer composition through copolymerization or by varying the concentration of chemical cross‐linkers used during synthesis.^[^ ^208^, ^215^, ^216^, ^217^ ^]^

Hydrogels are widely utilized in 4D printing because of their inherent swelling capability, which enables the creation of shape‐morphing structures.^[^ ^218^, ^219^, ^220^, ^221^ ^]^ Smart hydrogels are valued for their responsiveness to local stimuli such as changes in temperature, pH, and ionic strength or external factors such as electric fields and light.^[^ ^222^ ^]^ These stimuli alter the swelling ratio of a hydrogel, leading to volumetric or geometric transformations. To achieve complex deformations, such as bending, twisting, or folding, an internal swelling mismatch must be engineered. This can be accomplished by either a) the use of a single hydrogel with spatial variations in swelling behavior (induced by anisotropic fillers or variable crosslinking density) or b) the use of multimaterial hydrogel systems where different segments of the hydrogel respond differently to stimuli. Effective shape morphing also depends on several critical parameters, such as the volume fraction of the responsive material, the magnitude of swelling change upon stimulation, the actuation speed, and the mechanical robustness. Additionally, the hydrogel must maintain performance over multiple stimulus cycles without structural degradation or diminished responsiveness, ensuring durability for biomedical applications.^[^ ^223^ ^]^ 4D‐printed constructs have a wide range of applications, including sensors, soft actuators, controlled drug delivery systems, and in vitro models for cellular differentiation. However, enabling complex and programmable shape transformations in hydrogels demands the incorporation of designs such as nonhomogeneous geometries and multimaterial configurations and the precise engineering of conditions and pathways for stimulus application to ensure accurate and repeatable actuation.^[^ ^215^, ^223^, ^224^, ^225^, ^226^, ^227^ ^]^

The behavior of SMAs is governed by phase transitions between martensite (stable at lower temperatures) and austenite (stable at higher temperatures). On the basis of their cyclic behavior, SMAs are classified as one‐way, two‐way, or pseudoelastic.^[^ ^228^, ^229^ ^]^ One‐way and two‐way SMAs exhibit shape‐memory effects (SMEs) and can be programmed for reversible transformations, making them suitable for 4D printing.^[^ ^228^, ^229^ ^]^ In contrast, pseudoelastic SMAs recover their shape passively, without SME‐driven programming, and thus function more like elastic materials, limiting their relevance for 4D‐printed systems that require active and programmable morphing.^[^ ^228^, ^229^ ^]^

Liquid crystalline elastomers (LCEs) are highly programmable soft materials that merge the elasticity of lightly cross‐linked polymer networks with the stimuli‐responsive behavior of liquid crystals (LCs). Owing to the dynamic molecular orientation of mesogens (LC segments), LCEs can undergo substantial and reversible deformations when exposed to stimuli such as heat, light, electric or magnetic fields, humidity, or solvents. These changes are governed by mechanisms such as molecular reorientation, pitch modulation, or mesophase‐to‐isotropic phase transitions, without the need for water or energy sources.^[^ ^211^ ^]^ LCEs demonstrate exceptional anisotropic mechanical and optical responses, making them ideal for applications in soft actuators, artificial muscles, adaptive optics, soft robotics, energy absorbers, and active structures. For example, thermally responsive LCEs can produce reversible strains of up to 400% through phase transitions, and a typical LCE actuator strip can lift over 1000 times its own weight. This corresponds to a tensile stress near 300 kPa, a strain of ≈50%, and a specific work capacity of approximately 50 J kg^−1^, which are performance metrics that rival those of biological muscle tissues.^[^ ^208^, ^209^, ^210^, ^211^, ^212^, ^213^, ^214^ ^]^

##### Studies—Concept Studies

An implant that can change its rigidity properties as tissues heal and transfer load to the healing tissue, such as bone, would be highly important. It is known that rigid bone implants result in stress shielding depriving bone from physiological loading, consequently weakening the bone, loosening the implant, and increasing the risk of fracture. Therefore, the strength of self‐transforming implants can change with time, and changes in the properties of the healed bone will be a great achievement in the future. Changes in the shape of the implant will also be needed so that the implant can expand healing tissue or expand to fill in the gaps left by the remodeling of the healing tissue or in other situations, where the dynamics of the pathology being treated change over time, such as in the treatment of bone defects, where a marginal death of tissues can be absorbed and replaced by a dynamic implant that is installed when this void is not present.

The stiffness, which varies between either soft‐to‐hard or hard‐to‐soft, is an important property of devices. Low rigidity and good compliance are required by implants on/in soft tissues to reduce mechanical resistance, promote morphological adaptation, or accommodate organ micromotions during normal movement, whereas the surgical insertion process may require high rigidity for ease of handling. For example, temperature‐triggered soft‒rigid phase transition devices were fabricated with gallium and used as deep‐brain neural probes. The device remains rigid at the temperature of the operating room, and it softens following deep brain injection. This helps the implant accommodate micromotions occurring inside the skull during ordinary movement. The benefits of this approach are both its reduced lesion size and inflammatory glial responses compared with those of tungsten needles of the same dimensions, suggesting that the transformative approach is more amenable to deep embedding in soft tissue.^[^ ^197^ ^]^ In contrast, high rigidity is required by hard tissue implants for structural load bearing, whereas low rigidity and material compliance might be ideal for morphing and integrating into surrounding tissue. For example, biochemically induced variable‐stiffness actuators that can take different preprogrammed forms and change from soft to rigid by growing bone can be made. These materials can be utilized as morphing biological adhesives that are useful for the treatment of fractures or can be applied inside cavities to expand and initiate bone regrowth.^[^ ^189^ ^]^

An innovative climbing‐inspired concept was introduced in the form of a prototype that forms 3D twining electrodes, which can self‐climb onto peripheral nerves via smart shape memory polymers (SMPs) that can respond to body temperature.^[^ ^202^ ^]^ Designing 3D twining electrodes is performed by integrating stretchable mesh serpentine wires onto a flexible shape memory substrate.

The implants based on type 1 collagen have shown the ability to self‐expand from a crimped state to a tubular shape, which can potentially be used for vascular disease treatment.^[^ ^230^ ^]^

##### Studies—Material Studies

In one study, a tubular structure with multiple cells that can mimic blood vessels was fabricated via the SIRM technique via two elastic polydimethylsiloxane (PDMS) membranes.^[^ ^181^ ^]^ The top membrane is made of cured PDMS, which is stretched to produce stress, whereas the bottom membrane is a semicured PDMS membrane fabricated by spin‐coating uncured PDMS. The bottom layer acts as an adhesive layer that binds with the top layer during the curing process. The developed SIRM was covered with a PDMS mold embedded with microfluidic channels that can deliver different types of cells to the surface of the SIRM. The cell sheet rolls up with the SIRM to produce a layered wall of the tube, similar to vessel structures. By a simple rolling process, 2D membranes are transformed into 3D tubes that can mimic blood vessels. The shape of the tube is supported by the balance of internal stress and strain. Moreover, it reforms itself very quickly after the delivery of multiple cells to designated areas.

In another study, the same group fabricated self‐adjustable tubular scaffolds based on PCL and poly(lactide‐*co*‐glycolide) (PLGA) that can retain the shape of the scaffold during biodegradation.^[^ ^200^ ^]^ PCL‐PLGA multilayered tubes were fabricated via SIRM and microfluidic techniques. The inner PCL layer expanded, and the outer PLGA layer shrank to maintain the stability of the tubular structure. The tubular structure was able to self‐adjust and maintain the shape of the vessels even after degradation. This approach can be useful for designing biodegradable tubular scaffolds that can self‐adjust and maintain shape and architecture.

##### Studies—In Vitro Studies

Three types of cells, namely, endothelial cells, smooth muscle cells, and fibroblasts, were patterned on the surface of the SIRM. The cell‐covered SIRM begins to roll into a tube due to internal stress after one end of the SIRM is cut with a scalpel, and the cell sheet is layered as the tubular wall. To improve cell viability in long‐term cultures, bottom layers were fabricated with pillars. When they roll, they effectively separate the layers of the membrane, providing sufficient space for the medium to enter and help keep the cells alive. Even though some cells are injured during rolling, most cells inside the tubes were found to be alive after three days.^[^ ^181^ ^]^

The self‐adjusting and shape memory properties of PCL‐PLGA tubes were tested in vitro via incubation in cell culture media (human umbilical vein endothelial cells (HUVECs), smooth muscle cells (SMCs), and fibroblasts) and compared with those of tubes made of either PCL or PLGA.^[^ ^200^ ^]^ PCL‐PLGA tubes exhibited loose inner cavities at the initial stages of incubation, and after 21 days, they shrank, and all their layers merged into one layer. The inner diameter of the PCL‐PLGA tubes remained constant throughout the study, indicating self‐adjustment in this environment, whereas the PCL and PLGA tubes collapsed completely after 21 days because of the underlying shrinking and swelling of the polymers (Figure 4b). In the case of the composite (PCL‐PLGA blend), the PCL‐PLGA layers are more stable because the PCL component can hold the shape for a longer duration without degrading the PLGA layers. The difference in the degradation rates of PCL (slower) and PLGA (faster) was crucial for maintaining the durable mechanical support of the architecture of the tubes. The endothelial cell responses and functions of shape‐morphing scaffolds with nanofibrous inner layers made of electrospun PCL/GelMA membranes were studied. Compared with the electrospun PCL membrane, this layer supported homogeneous endothelial cell attachment, the formation of biomimetic cell–scaffold interactions, and cell–cell interactions.^[^ ^180^ ^]^

In vitro studies revealed that HAp‐GS3 (GS3 indicates 2.5 wt.% GS), which had the highest drug loading, exhibited high antibacterial ability against *E. coli* and *S. aureus*, even after five repeated tests, whereas pristine HAp displayed no antibacterial ability.^[^ ^196^ ^]^ Furthermore, the porous structure of HAp provided ample space for bacterial proliferation. These results confirmed the sustainable antibacterial ability of the self‐adaptable HAp‐GS material.

Cytocompatibility studies on self‐expandable collagen scaffolds performed using human endothelial cells and human smooth muscle cells revealed that cells were present on both the luminal side and outside of the scaffold; however, no penetration was observed in the interior regions of the scaffolds.^[^ ^231^ ^]^

##### Studies—In Vivo Studies

The in vivo conforming behavior of the shape‐changing and softening organic electronics was observed. An initially rigid, planar electronics with an SMP coating was implanted under the skin of a rat. Over the first few hours after surgery, fluid uptake by SMP led to plasticization, triggering softening (from >1 GPa to ≈80 MPa). The initially planar devices softened and conformed to the biological environment and withstood 24 hours of fluid exposure and deformation associated with implantation in living tissue while maintaining electrical properties without significant degradation (Figure 4c).^[^ ^183^ ^]^

Before implantation, the twining electrode was flat, making it convenient for handling and implantation. Following implantation next to the vagus nerve of the rabbits, the twining process of the electrode was driven by 37 °C saline. It underwent a self‐climbing process similar to what is seen with twining plants. Accordingly, the twining electrode can conformally contact the vagus nerve, forming a good interface even under extreme deformation forces (Figure 4d).^[^ ^184^ ^]^

The benefits of transformative electronics, such as neural probes, were observed after one month of implantation in the mouse brain. The device remained rigid at the temperature of the operating room, but upon application in deep brain tissue, it softened to match local tissue.^[^ ^197^ ^]^ Compared with nontransforming tungsten needles of the same dimensions, the transformative neural probe had both a reduced lesion size and inflammatory glial response, suggesting that the transformative approach is more amenable to deep tissue embedding. The devices remained intact and functional for at least six weeks following deep brain implantation.^[^ ^197^ ^]^

In vivo studies conducted in rabbits demonstrated the ability of self‐adjusting PCL‐PLGA artificial blood vessels to maintain shape and structure.^[^ ^200^ ^]^ Artificial vascular replacement of the left carotid artery with PCL‐PLGA tubes as artificial blood vessels was performed, and the flow was monitored via Doppler ultrasound. Three months after surgery, the implant allowed continuous blood flow with no significant change in the diameter of the tubes, whereas blood flow was obstructed in the control PCL and PLGA tubes after three months because of their clog. PCL‐ and PLGA‐only tubes undergo severe structural damage, leading to excessive tissue hyperplasia, whereas in the composite PCL‐PLGA tubes, the shrinkage of the PLGA‐outer layer and well‐preserved PCL inner layer contributed to structural maintenance of the tubes and smooth transition from artificial blood vessels to fully remodeled native blood vessels.

##### Studies—Clinical Studies

To date, no clinical studies have been reported.

##### Summary

The development of such well‐controlled, complex structures that morph to predetermined shapes or self‐change their mechanical properties has potential for applications in tissue engineering, drug delivery, sensing, soft robotics, and specific surgical procedures. Innovative techniques such as the use of twinning electrodes can greatly reduce nerve injury caused by mechanical mismatch and surgical implantation. The development of self‐adaptive implants with a sustainable response provides an alternative novel approach for addressing problems such as implant‐associated infection. The self‐expandable implants can be crimped to a fraction of their original size and re‐expands upon exposure to fluids such as blood, which makes them suitable for application via minimally invasive procedures.^[^ ^232^ ^]^ No reports of material studies were identified. In vitro studies demonstrated antibacterial ability against *E. coli* and *S. aureus*. In vivo studies demonstrated antibacterial responses and self‐adaptive drug release. In the future, these implants may have potential for use as biological vascular implants.

#### 2.2.4 Self‐Awareness

Self‐awareness can be defined as an implant's ability to sense and monitor its orientation, location and status when it is implanted in the body.^[^ ^16^, ^17^ ^]^ Self‐awareness is very important for an implant to be able to “feel” its surroundings and make appropriate adjustments and responses. To achieve self‐awareness, many capabilities should, however, be possessed by the implant. These include sensing, self‐powering, and self‐monitoring.^[^ ^16^ ^]^ To date, this could be achieved by the assembly of meta‐tribomaterial sensors and nanogenerators. Self‐aware implants have been developed with the vision of being a part of personalized implants and aiding in detecting and reducing complications in the future.

##### Mechanism

Self‐awareness can be achieved by using composite metamaterials known as self‐aware composite mechanical metamaterials (SCMMs), which can transform mechanical metamaterials into nanogenerators and active sensing tools. Self‐aware materials can sense and self‐program, using their constituent components. In addition, they can also be self‐powering. The system is an integration of self‐recovering snapping microstructures consisting of topologically different triboelectric materials that can provide the functions of the tribomaterial system. Self‐aware implants are structure dominated; hence, on the basis of the target application, their geometry (shape, size, and stiffness) can be tuned by modifying the number and deformation sequence of auxetic cells, integrated microstructures, and layer material.

**Figure 5:**
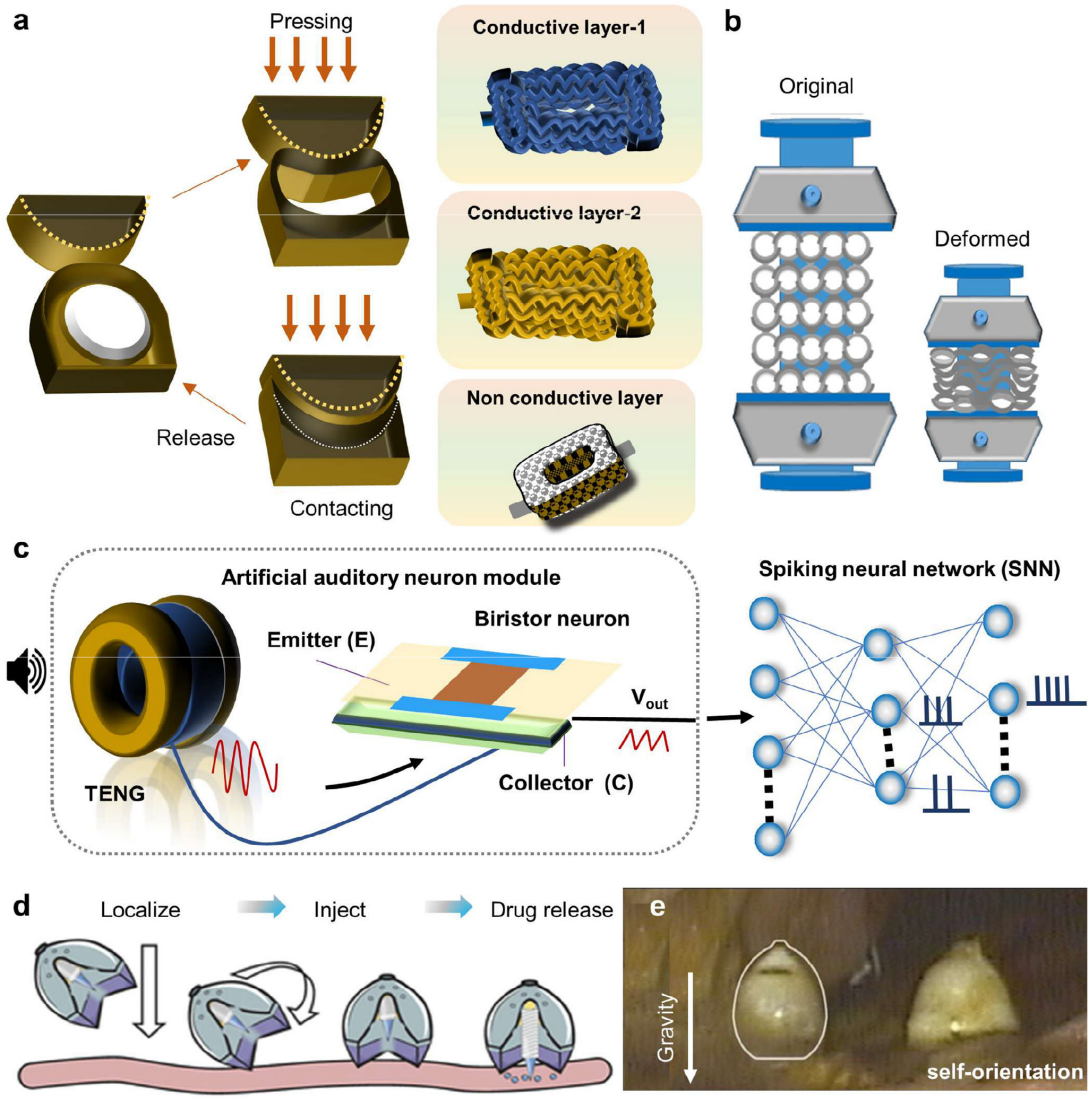
Self‐aware implants. An illustration of a nanogenerator interbody fusion cage implanted during spinal fusion surgery, showing a) components of a self‐aware cage implant, b) a 3D printed self‐aware composite mechanical metamaterial (SCMM) at the original and deformed states under cyclic loading, c) a self‐aware artificial auditory system consisting of a triboelectric nanogenerator (TENG) and biristor neuron that can generate spikes for neural processing with a spiking neural network (SNN), d) an illustration of a self‐orientating millimeter‐scale applicator (SOMA) showing its orientation in the stomach toward the gastric wall, and e) an image of the SOMA device showing the self‐orientation of the SOMA device in a porcine stomach, following its administration and agitation of the abdomen via 180° rotations and 30° tilts. a) Reproduced with permission.^[^ ^16^ ^]^ Copyright 2022, Wiley‐VCH; b) Reproduced with permission.^[^ ^17^ ^]^ Copyright 2021, Elsevier; c) Reproduced with permission.^[^ ^239^ ^]^ Copyright 2023, Elsevier. d,e) Reproduced with permission.^[^ ^238^ ^]^ Copyright 2019, AAAS.

Recently, this mechanism was studied for the production of self‐aware spinal fusion cage implants (**Figure** **5**a).^[^ ^16^ ^]^ Implants integrate conductive and nonconductive triboelectric layers, which are semicircular‐shaped curved elements that can provide support, as well as elastic snapping before and after deformation. During spinal movement, an electrical output is generated by the contact of conducting and nonconductive layers of the cage, with the voltage signal being proportional to the forces applied to the structure. The mechanism is based on a relative healing approach, where the voltage signal generated in the cage is compared with the signals in the previous stage, which is considered the reference baseline. The implant can thus serve as a sensor and energy harvesting (EH) mechanism.

Compression loading tests revealed that when the rings of a synthetic spinal model and fusion cage become stiffer, they tend to carry more weight, which reduces the strains inside the cage, resulting in a lower voltage. The decrease in voltage, in turn, indicates progress in the healing process. The proposed self‐aware technology is a new path for monitoring the healing process. Furthermore, innovative self‐aware implants can be fabricated using a wide range of metals, such as Ti and Au; bioresorbable polymers, such as polylactide (PLA) and PLGA; and bioresorbable metallic (magnesium) materials with triboelectric charging.

Another strategy was adopted to develop a self‐aware artificial auditory neuronal part of a cochlear implant that reduces power consumption via a spiking neural network (SNN).^[^ ^235^ ^]^ The SNN‐based auditory system possesses self‐aware sensing capability for sound signals. The system comprises a triboelectric nanogenerator (TENG) as the auditory sensor and a bistable resistor (biristor) as the spiking neuron (Figure 5c). When sound pressure was applied to the TENG, alternating current (AC) was generated and transferred to the biristor neuron as the input; subsequently, the biristor neuron converted the current input to voltage as the output for the SNN. The system was tested via a musical instrument for detecting sound pitch with measured spiking characteristics, and it was able to distinguish two different sound pitches originating from a piano.

These functionalities are achieved via the integration of nanoenergy harvesting TENG mechanisms and mechanical metamaterial design paradigms. The multifunctional metamaterial systems act as TENGs capable of generating electrical signals when stimulated by the application of mechanical excitations. The electrical signal can then be used either for sensing the applied force or for storing it to empower sensors and embedded electronics.

##### Studies (Explanatory Examples)—Concept/Design

Self‐aware implants are still in the very early stage of development where the concept is being tested. For example, investigators have developed an SCMM‐based self‐sensing and self‐powering cardiovascular stent and cardiac shock absorber.^[^ ^17^ ^]^ The concept of a “self‐aware composite mechanical metamaterial” was proposed by Barri et al.^[^ ^236^, ^237^ ^]^ to enable the creation of a new generation of multifunctional and metamaterial implantable devices that can respond to their environment, power themselves, and self‐monitor their condition.

##### Studies (Explanatory Examples)—Material Studies

The design of self‐aware implants depends on many parameters, including the type of material, thermal‐mechanical stability, and cost‐effectiveness. Although the use of natural materials is appealing, because of their limitations in terms of energy conversion, integration in implants, and mechanical properties to sustain loading, synthetic materials are used for the fabrication of self‐aware implants. Synthetic metamaterials can be multifunctional and can be used to develop composite self‐aware structures that can convert metamaterials into nanogenerators and sensing devices.^[^ ^17^ ^]^

Composite mechanical meta‐tribomaterial systems can also be developed using parallel snapping curved segments that exhibit mechanical self‐recovery behavior.^[^ ^17^ ^]^ To develop this system, a metamaterial was fabricated using thick horizontal and vertical elements and thin curved parts. The 2D structure of the snapping composite metamaterial consists of two conductive layers embedded with a thicker dielectric layer (Figure 5b). Uniaxial loading between 15 and 45 N applied to the 3D‐printed metamaterial structure resulted in a large deformation due to the discrepancy between the snapping and supporting components. However, when the force was applied vertically to the center of the curved beams, the semicircular‐shaped segment shells were mechanically deformed (Figure 5b). When the SCMM concept was applied to design shock absorbers, continuous measurements of the applied forces and energy harvesting were taken from the external mechanical excitations. Although the SCMM concept has some innovative advantages for designing self‐aware energy harvesting metamaterials, the actual implementation of this concept still needs to be explored further to optimize the setup, durability, and robustness of the material and viability for commercial fabrication.

The feasibility of the SCMM mechanism for designing multifunctional systems such as cardiovascular stents and shock absorbers was demonstrated.^[^ ^17^ ^]^ To achieve this goal, a layered meta‐tribomaterial system consisting of integrated microstructures of topologically different triboelectric materials to act as a TENG and sensing system was used.^[^ ^17^ ^]^ The conductive layer was made of carbon black, whereas the dielectric layers were made of PLA and polyurethane. The deformation mode of the integrated microstructure of the implant generates contact electrification when the two surfaces of the SCMM structure undergo periodic changes due to mechanical excitation. The two layers of the system act as conductive and dialectic layers that collect positive and negative charges. It is presumed that when the SCMM structure is loaded, the charge remains on the surface of the dielectric layer, which results in a static electric field and a potential difference between the conductive layers. The snapping SCMM structures undergo periodic deformation under compressive loading, which results in contact electrification between the conductive and nonconductive surfaces. By unloading the structure, a potential difference occurs between the conductive layers. Consequently, more contact takes place between the conductive and dielectric layers, which generates a high electrical output. The generated electrical output signals provide active sensing, while the generated electrical energy can be harvested and stored to empower sensors and electronics of the implant. Moreover, metamaterial implants with the TENG mechanism provide good mechanical stability to the implant.

Inspired by the leopard tortoise, which has curvature to position itself toward the center of mass and a strong upper shell architecture that anchors the self‐orientation toward the upright position, an ingestible self‐orientating millimeter‐scale applicator (SOMA) was developed.^[^ ^238^ ^]^ It comprises a monomonostatic body that can independently position itself in the gastrointestinal tract, insert drug‐loaded milliposts into the stomach, and then travel down to exit (Figure 5d). Low‐density PCL and high‐density stainless steel were used to produce the low center mass needed for SOMA for the self‐orientation of the device. A stainless‐steel spring was used as a power source considering the limited space and force generation along the single axis.

##### Studies (Explanatory Examples)—In Vitro Studies

In vitro studies demonstrated self‐awareness of the self‐oriented SOMA implant.^[^ ^238^ ^]^ When the implant was placed on a tilt shaker running at 50 rpm with an excursion of 15°, the SOMA did not tilt more than a single degree and oriented itself quickly from 30° and 135° angles.

##### Studies (Explanatory Examples)—In Vivo Studies

Experiments in swine stomachs (ex vivo) and fasted (in vivo) swine revealed that the mass distribution of SOMAs did not affect the orientation of the implants in the stomach and that compared with the virgin PCL‐based device, which was oriented only 50% of the time, the SOMA‐based device oriented 100% of the trials. Microcomputed tomography (micro‐CT) images from in situ and *ex vivo* experiments demonstrated the insertion of the implant milliposts into the stomach (Figure 5e). The milliposts were inserted into swine tissue 7 mm long when a force of 1 N was applied, and ≈0.3 mg of insulin was delivered. No signs of damage to the stomach were observed via endoscopy performed one week post‐injection. Histological studies confirmed that the device can deliver milliposts without injuring other muscular layers of the stomach.

##### Studies (Explanatory Examples)—Clinical Experiments/Trials/Routine Use

To our knowledge, no clinical studies on this topic have been reported thus far.

##### Summary

Self‐aware implants constitute an innovative concept that provides the next generation of implants, although the experiments conducted are limited to spinal implants using spine and human cadaver spine models, cardiovascular stents, and cardiac shock absorbers. A self‐orientation millimeter scale applicator (SOMA), which autonomously inserts a drug‐loaded millipost into the stomach, can be considered one step forward in achieving self‐orientation, yet there are certain hurdles that need to be addressed. For example, there is a limited deliverable dose and an appropriate insertion technique, as we do not have proper control over the depth and width of the millipore penetration, which needs to be addressed in future research. Although studies have shown the orientation of SOMAs in some fluids, more experiments need to be conducted using fluids with different viscosities and under varying conditions, such as temperature, time, and internal pressure. In vitro studies have thus far been conducted only to test the self‐awareness of SOMA implants, and more experiments need to be conducted on other self‐aware implants. More in vivo studies on large animals are needed before clinical use can be pursued. Other aspects, such as mechanical stability and biocompatibility, need investigation. Studies of economic aspects and scale‐up should be carried out.

#### 2.2.5 Self‐Actuation

The self‐actuation property of an implant is the action taken by the implant, e.g., the release of loaded cargo following exposure to a specific trigger, without the need for external control. Self‐actuation could also include changes in the implant shape, mechanical properties, position, electromagnetic properties, biomaterial surface status, or other actions. Instead of externally aided actuation, self‐actuation can be a better strategy that can be developed and integrated into future autonomous implants.^[^ ^240^ ^]^ The purpose of having a self‐actuation implant is to start an action without the need for external instruction, but only to specific triggers. For example, a self‐actuating implant would release an antimicrobial agent in response to infection.

##### Mechanism

**Figure 6:**
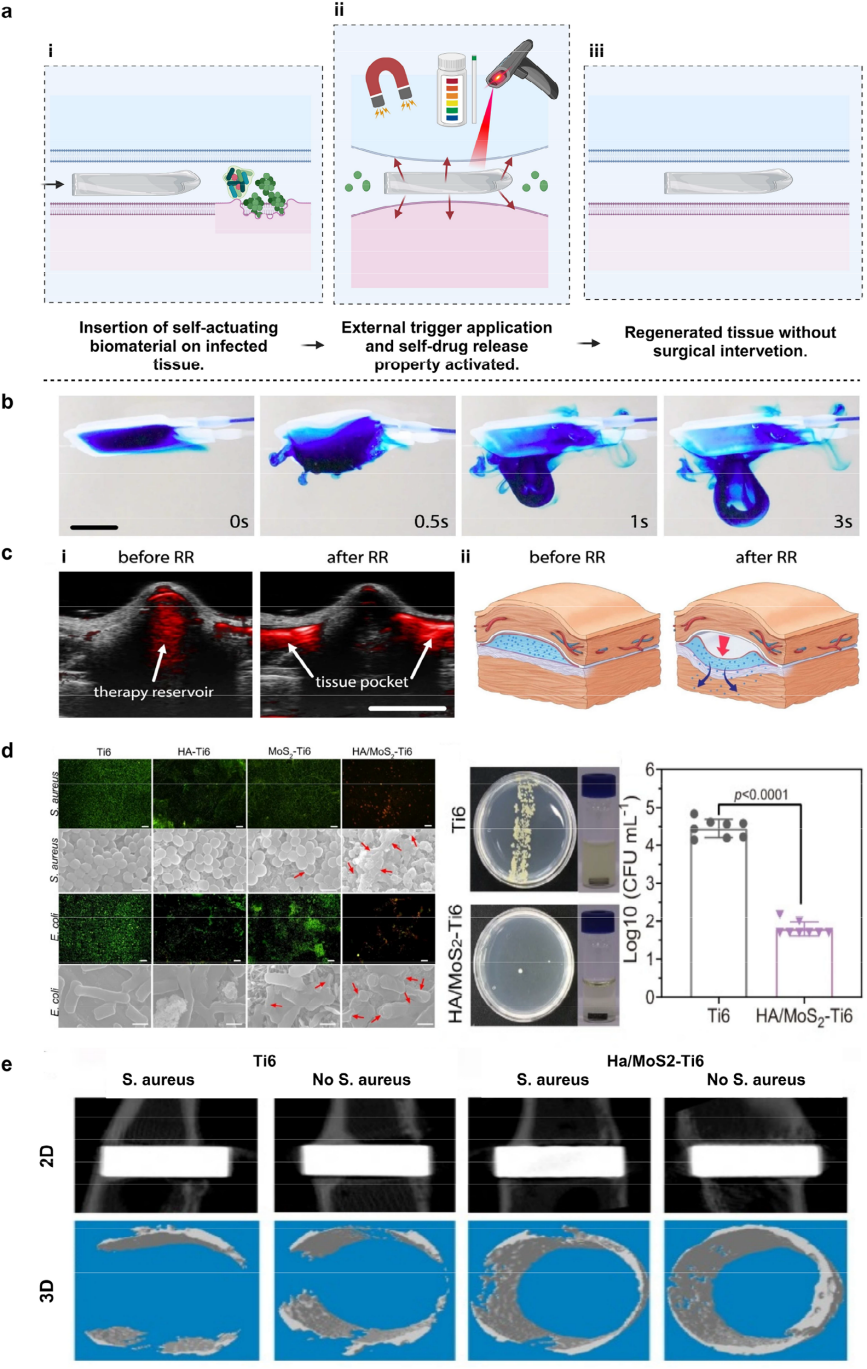
Self‐actuating/activating implants. a) Mechanisms of self‐actuation. a, i) Self‐actuating implants used to treat contaminated tissue. a, ii) Activation of drug release from the self‐actuating implant triggered by changes in the local pH, photoexposure or magnetic field. a, iii) Tissue regeneration by a self‐antibacterial release device. b and c) Examples of designs and self‐activation. b) Drug release by a self‐actuating implant demonstrated using methylene blue. c, i) Photoacoustic images demonstrating the release of the drug (red area) from a self‐actuating platform implanted in a rodent model, showing the subcutaneous transfer of the drug from the implant drug reservoir to the tissue pockets of the animal. c, ii) Mechanisms of drug release and self‐actuation presented by the developed system. The implanted platform self‐actuates compression on the above membrane, releasing the drug from the lower membrane to the tissues caused by the foreign body response underlying the platform. Legend: RR: rapid release. d,e) Application studies (in vitro and in vivo). d) In vitro application of implant surfaces with self‐antibacterial properties. The self‐activated antibacterial effect caused by the HA/MoS2‐Ti6 coating reduced *E. coli* and *S. aureus* survival, as demonstrated by qualitative images (microscopies—LIVE/DEAD and SEM techniques) and quantitative colony counts (graphs). e) In vivo application of implants with self‐antibacterial surfaces in rat bones. The quality of bone integration in contaminated or noncontaminated environments, as shown by radiographs and micro‐CT, was better for bone regeneration and bacterial combat than for implants without an innovative surface. Figure a was created via Biorender. b–e) Reproduced with permission.^[^ ^21^, ^247^ ^]^ Copyright 2021, 2022, Springer Nature – under a Creative Commons Attribution 4.0 International License.

Actuation takes place after changes in the properties of an implant are triggered by a specific stimulus, e.g., a low pH in the surrounding environment, resulting in changes in its properties, including chemical activity, porosity, and mechanical structure that leads to the release of its cargo, e.g., the release of antibiotics in response to a developing peri‐implant infection^[^ ^241^ ^]^ (**Figure** **6**a(i–iii)). Some of these stimuli result from the interaction between the implant surface and cell membranes,^[^ ^241^ ^]^ triggering physicochemical reactions at the implant‐tissue interface.

In one example, the transfer of electrons between the coating material (HAp/molybdenum sulfide, HAp/MoS_2_) and bacteria that may come in contact with the implant leads to self‐activation of the antibacterial agent included in the coating, i.e., HAp/MoS_2_.^[^ ^21^ ^]^ In another surface mechanism based on a superhydrophobic star‐shaped response platform, superior antiadhesion self‐actuation was demonstrated in experiments with *E. coli* and *S. aureus* generated by a self‐actuated Cassie‒Baxter wetting state, which substantially reduced adhesion to bacteria.^[^ ^242^ ^]^ When they take up water, hydrogels can generate a difference in the hydrostatic pressure in certain devices that can have self‐bending properties. For example, self‐bending electrodes in CIs can be applied to induce electrode contact with nerve cells of the inner ear.^[^ ^243^ ^]^ Hydrogels can also be used to develop self‐folded devices for the engineering of functional cardiac tissue.^[^ ^244^ ^]^ Varying biomaterial stiffness can be used to stimulate self‐regeneration of tissues.^[^ ^7^ ^]^ Moreover, changes in implant architecture can be achieved by the incorporation of fungi into implants due to the expansion of fungi promoted by favorable environmental conditions.^[^ ^19^ ^]^

##### Studies—Concept/Design Studies

Similarly, a multifunctional hydrogel coating on phthalazinone (PPENK) was proposed for biomedical implants. The multifunctional gelatin containing black phosphorus promoted self‐activating antibacterial properties under photothermal mediation associated with osteogenic features by activating the locally controlled release of loaded doxorubicin and the generation of reactive O_2_ species (ROS) to combat bacteria.^[^ ^245^ ^]^ In combination with *S. aureus*, a PCN‐222 metal organic framework incorporating bismuth nanoparticles was proposed as a rapid therapy for infected wounds. The physicochemical status of this biomimetic nanozyme is capable of inducing a difference in the charge potential of the bacterial membrane, promoting self‐driven electron transfer at the interface, which is suggested to disturb and impair bacterial metabolism, suggesting potential for the design of self‐antibacterial biomaterials.^[^ ^246^ ^]^ To control the foreign body response and extend the efficacy of drug‐releasing devices, a dynamic actuation approach was pursued by using synergistic soft robotic abilities: intermittent actuation and controlled actuation (Figure 6b,c).^[^ ^247^ ^]^

Self‐actuating preprogrammed materials that can vary their stiffness actuators to influence tissue healing and regeneration, e.g., of the bone, can be useful biomedical implants. To demonstrate this, a hybrid device was developed. The device changes from soft to stiff material by the bone mineralization process in its internal layer (made of polypyrrole). The hybrid device was produced of alginate functionalized with cell‐derived plasma membrane nanofragments, which promoted this biological process.^[^ ^27^ ^]^

##### Studies—Material Studies

The mechanotherapeutic function of applying cycling stimulation for 5 min every 12 h to preserve long drug release of the system was tested in a human cadaver model via minimally invasive percutaneous implantation at the *transversus abdominis* muscles of a soft actuatable platform with drug release features that are easy to manipulate in a human‐scale device, which is promising for clinical use, e.g., in the treatment of fibrosis and type 1 diabetes.^[^ ^247^ ^]^

##### Studies—In Vitro Studies

The antibacterial efficacy of the self‐activating antibacterial HAp/MoS_2_ coating of Ti6Al4V (Ti6) implants against *S. aureus* and *E. coli* was examined in vitro (Figure 6d).^[^ ^21^ ^]^ This leads to a reduction in the count of methicillin‐resistant *S. aureus* (MRSA), which is attributed to MoS_2_.^[^ ^248^ ^]^ The combination of printed hydrogels incorporated with living organisms (fungal mycelium) led to the development of self‐cleaning 3D scaffolds when supported by nutrients and water.^[^ ^18^ ^]^ The growth of these fungi in the hydrogel expands its layers and changes the shape of the biomaterial.

##### Studies—In Vivo Studies

Experiments performed in rats revealed that, compared with noncoated implants, *S. aureus‐*contaminated HAp/MoS_2_‐Ti6‐coated implants had reduced bacterial colonization after 14 days of implantation.^[^ ^21^ ^]^ There were also fewer neutrophils around the coated implants than around the noncoated implants (Figure 6d,e). A self‐aligning intraocular pressure measuring device that employs gold‐coated neodymium‐iron boron (NdFeB) micromagnet sleeves over fibers, which generate a strong magnetic field that enables the fibers to maintain alignment,^[^ ^249^, ^250^ ^]^ was tested in rabbits and was found to provide stable measurements at constant hydrostatic pressure in the eyes of rabbits.^[^ ^251^ ^]^

##### Studies—Clinical

To date, no clinical studies on self‐actuating implants have been reported.

##### Summary

Although self‐actuating systems have been developed in various forms, the technology is still in its infancy, and studies remain limited to lab tests, with a few using in vitro cell culture, one using human cadaver implantation at the abdominal muscles to investigate clinical translatability and a couple using animal experiments. Technology remains to be properly evaluated both in vitro and in vivo for biocompatibility and functionality and validated using comparable existing methods if such conventional therapies are used at all. Because of the complexity of the system, multidisciplinary teams are needed. A close eye should be paid to this emerging technology.

#### 2.2.6 Self‐Powering

A self‐powering implant is an implant whose power is independent of batteries or the external supply of energy. Although there have been advances in the development of implantable batteries^[^ ^252^ ^]^ problems with a continuous power supply for integrated systems with more complex systems that require more power remain.^[^ ^253^ ^]^ In addition, conventional lithium‐ion batteries may contain toxic substances in both electrodes and electrolytes. For long‐term service, batteries also need to be surgically replaced periodically, which imposes a considerable economic and health burden. External power using wire was developed, but it carries the risk of developing infection. Alternatively, external wireless powering was explored by using inductive power transfer,^[^ ^254^ ^]^ radiofrequency irradiation,^[^ ^255^ ^]^ acoustic power transfer,^[^ ^256^ ^]^ optical power transfer,^[^ ^257^ ^]^ magnetoelectric power transfer,^[^ ^258^ ^]^ and capacitive power transfer.^[^ ^259^, ^260^, ^261^, ^262^ ^]^ However, this strategy is associated with challenges related to performance,^[^ ^263^ ^]^ tissue absorption,^[^ ^264^, ^265^ ^]^ the receivers required to capture sufficient power,^[^ ^266^ ^]^ etc. Therefore, extensive research has been carried out into designing self‐powered implants in the past few years,^[^ ^16^, ^32^, ^267^ ^]^ which relies on EH to overcome problems associated with batteries and external powering.

##### Mechanism

**Figure 7:**
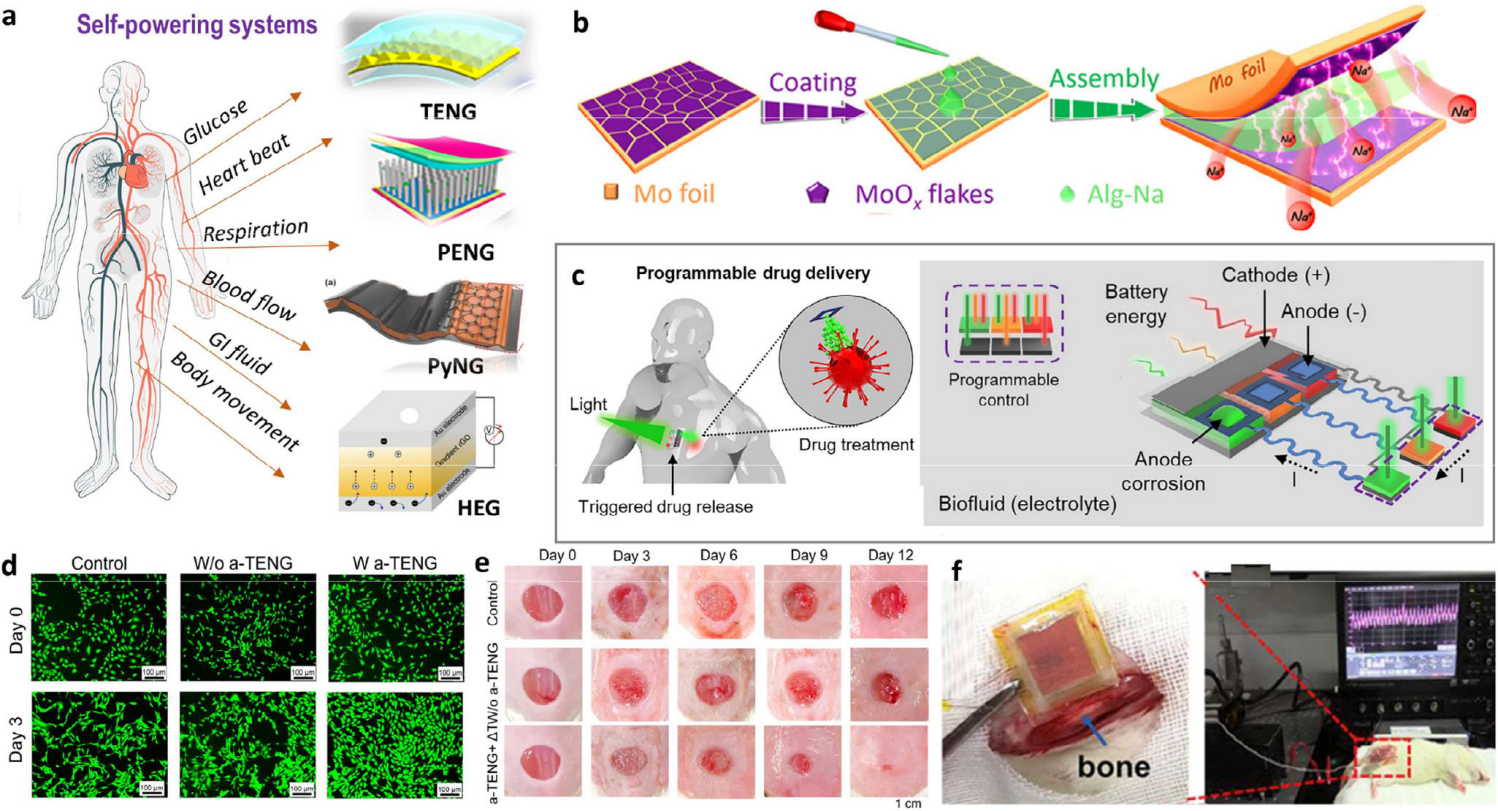
Self‐powering. a) Power sources generated by body movement, flow, heat, or chemical changes and energy harvesting systems such as triboelectric nanogenerator (TENG), piezoelectric nanogenerator (PENG), pyroelectric nanogenerator (PyNG) and hygroelectric nanogenerator (HEG) devices utilized for conversion. b) Preparation of fully biodegradable supercapacitor implants by a sandwich construction of Mo foil (current collector), MoO_x_ flakes and Alg‐Na gel electrolyte. c) Schematic of self‐powered programmable release of drugs and a diagram showing the metal gate structure that serves as the anode of the battery system, and their light‐controlled electrochemical corrosion leads to controlled opening of drug reservoirs, therefore providing a basis for programmable release of drugs. The electrical resistance of the phototransistor decreases, and thus, short circuits occur in the electrochemical cell formed by the anode and cathode with surrounding biofluids as the electrolyte. The process initiates corrosion of the gate and opening of the underlying drug reservoir. Optical filters placed on top of the phototransistors create a wavelength‐selective response. d) Example of an in vitro study: Fluorescence images showing enhanced proliferation of fibroblasts electrically stimulated by TENGs for 3 days. e) Example of an in vivo study: Self‐powered treatment for the healing of *S. aureus*‐infected wounds. Images showing the wound contraction areas. The TENG groups presented an ∼ 32% relative wound closure area, which was approximately three times lower than that of the normal group after day 12. f) Example of the in vivo study: A flexible TENG was implanted on the surface of the femur region of a rat, and the output measurement process involved the daily movement of the rat by pulling the leg of the rat via a linear motor. b) Reproduced with permission.^[^ ^291^ ^]^ Copyright 2021, Science; c) Reproduced with permission.^[^ ^288^ ^]^ Copyright 2023, PNAS; d,e) Reproduced with permission.^[^ ^290^ ^]^ Copyright 2023, Science; f) Reproduced with permission.^[^ ^284^ ^]^ Copyright 2019, Elsevier.

Self‐powering can be achieved via EH. EH is a fundamental enabler of several capabilities of autonomous implants, including actuation, wireless communication, and sensing. Therefore, EH represents a core component for the development of autonomous implants. EH can be achieved by harvesting energy from the surroundings, such as energy generated by movement, flow, heat, or chemical changes (**Figure** **7**). Harvesting energy generated by body motion includes leveraging muscle contraction/relaxation (mechanical energy) and blood circulation (hydraulic energy). The chemical energy resulting from salts/glucose, etc., can also be converted into electrical energy.^[^ ^268^ ^]^ On the basis of the characteristics of energy forms, appropriate energy harvesting systems such as TENGs, piezoelectric nanogener (PENGs), pyroelectric nanogenerator (PyNG), hygroelectric nanogenerator (HEG) and electromagnetic power devices are utilized for conversion.^[^ ^262^, ^269^, ^270^ ^]^ For example, mechanical energy resulting from the movement of the heart, lung and diaphragm can be harvested via PENGs or TENGs to provide self‐powering of the implant.^[^ ^271^, ^272^ ^]^ The energy harvesting ability of TENGs relies on triboelectrification and electrostatic induction effects between two friction layers.^[^ ^273^ ^]^ The working mode and linkage of external circuits can be divided into four types: vertical contact separation mode, lateral sliding mode, single electrode mode, and freestanding triboelectric layer mode. The vertical contact separation type is widely used for self‐powered implant systems.^[^ ^274^ ^]^ Other mechanisms, such as electromagnetic induction^[^ ^275^ ^]^ and electromagnetic generators (EMGs), have also been suggested for energy harvesting. Recently, the use of flexible NdFeB magnets was shown to convert the mechanical energy associated with finger movement into electrical energy and power wearable sensors.^[^ ^275^ ^]^ Piezoelectric nanogenerators (PENGs) are another potential energy conversion device that utilizes the piezoelectric effect.^[^ ^276^ ^]^ When an external force is applied to piezoelectric nanomaterials, the internal electric voltage difference generates tension on materials although electric dipole moments.

##### Studies—Concept Studies

The concept of a self‐powered smart knee implant that generates power by the frictional sliding of two ridge‐shaped surfaces when a person walks the implant was presented.^[^ ^20^ ^]^ Sliding occurs as a result of the transfer of electrons from one surface to another and provides self‐powered charge to the implant. Other concepts associated with self‐powered sensors,^[^ ^277^ ^]^ actuators,^[^ ^278^ ^]^ human‐powered devices^[^ ^279^ ^]^ and others have been reported.^[^ ^32^ ^]^ In these concepts, implantable micro/nanosystems are self‐powering units that can operate for a long time within the body without the need to replace batteries, resulting in the harvesting of biomechanical energy from the body itself.

##### Studies—Material Studies

The first piezoelectric nanogenerator was based on zinc oxide (ZnO) nanowires (NWs), which convert mechanical energy into electrical energy.^[^ ^276^ ^]^ The mechanism of power generation is dependent on the coupling of the piezoelectric and semiconducting properties of ZnO. The nanogenerator was able to harvest energy from the environment and can be developed into a self‐powering unit. The ZnO nanowires were grown on an aluminum oxide (Al_2_O_3_) substrate, with Au particles used as a catalyst, via the vapor‒liquid‒solid (VLS) process.^[^ ^280^ ^]^ NW arrays were grown on an atomic force microscope tip in contact mode. The coupling of the piezoelectric and semiconducting properties of ZnO created a strain field and charge separation across the NW as a result of its bending. The rectifying characteristics of the potential energy barrier formed between the metal tip and the NW led to power generation with an efficiency of 17–30% for a cycle of resonance. The study revealed that by changing the size of the NW array, the power generated can drive small single nanotube‐based devices. Once the resonance of an NW array can be achieved to output the piezoelectric‐converted power generated in each cycle of vibration, a significantly strong power source becomes possible for self‐powering implants. In 2010, Li et al. first developed an implanted flexible PENG using two‐end bonded ZnO nanowires.^[^ ^281^ ^]^ Later, a self‐powering cardiac pacemaker was produced by harvesting the natural energy of heartbeats with a rigid PENG made of an elastic skeleton of a polyethylene terephthalate (PET) sheet, and two piezoelectric composites consisting of a piezoelectric layer, beryllium‐bronze foil, and Cr/Au electrodes were reported.^[^ ^282^ ^]^ More recently, a battery‐free heart pacemaker powered by a flexible PENG was developed. The PENG is composed of composite nanofibers of poly(vinylidene fluoride) (PVDF) and a hybrid nanofiller made of zinc oxide (ZnO) and reduced graphene oxide (rGO).^[^ ^283^ ^]^

A self‐powered electrical stimulator for inducing bone formation and healing was built using a TENG and a flexible interdigitated electrode with PET as the substrate and packed with PDMS.^[^ ^284^ ^]^ In another study, a self‐rechargeable cardiac pacemaker system with an in vivo TENG (I‐TENG) that works on the basis of body motion and gravity was developed.^[^ ^285^ ^]^ Gravity was achieved via the use of amine‐functionalized poly(vinyl alcohol) and perfluoroalkoxy groups as triboelectric materials. The maximum volume power density was 4.9 µWRMS cm^−3^ (at ≈10 MΩ), and the powder was harvested at ≈144 mW in preclinical animal experiments.

Bulk nanogenerators have limited utility because of their incongruent contact with the surface of organs such as the brain, lung, and heart; thus, they are unsuitable for harvesting the minute movements of internal organs, and a flexible nanogenerator is more suitable for energy harvesting on the organ surface. Such a flexible triboelectric active sensor (I‐TEAS) was fabricated by employing a multilayer thin‐film structure composed of triboelectric layers, electrodes, and spacers that were encapsulated with multilayer biocompatible materials, which provided real‐time and continuous monitoring functions for various physiological and pathological signs by sensing the biomechanical motion of intracorporeal organs.^[^ ^286^ ^]^ Another study reported a self‐powered flexible and implantable electrical stimulator consisting of a flexible TENG consisting of two friction layers of aluminum (Al) and polytetrafluoroethylene (PTFE) films, a rectifier and a flexible interdigitated electrode, which could be driven by daily movement to maintain bone homeostasis.^[^ ^284^ ^]^

In a recent study, a wearable self‐powered wound dressing was fabricated by integrating the thermocatalyst bismuth telluride (Bi_2_Te_3_) and a mechanical energy harvesting layer of chitosan‐coated carbon fiber fabrics (CFFs), which can be triggered by diverse stimuli (mechanical motion and temperature gradient) and can provide on‐demand treatment for both normal and infected wounds.^[^ ^269^ ^]^ The dressing structure was composed of two CFF‐based electrodes coated with a chitosan hydrogel on the exterior side. CFFs served as the substrate and electrode layers to maintain conformal contact with the wound, whereas the chitosan hydrogel was used as the external layer because of its biocompatibility and ability to retain moisture. The dressing was also triggered to generate a local electric field around the wound by connecting the integrated electrodes to an arch‐shaped TENG.

In a recent study, biodegradable supercapacitor implants were developed from molybdenum oxide (MoO_x_) flakes, which were grown in situ on water‐soluble Mo foil and sodium alginate gel (Alg‐Na) electrolytes via an electrochemical oxidation approach (Figure 7b).^[^ ^287^ ^]^ Anodized MoO_x_ flakes formed by applying an alternating voltage between 0 and 1 V are firmly adhered to the Mo foil current collection, which results in low contact resistance and alleviates the exfoliation of active materials during the electrochemical reactions. During continuous electrochemical cycling, the interconnected MoOx flakes form cracks filled with Alg‐Na gel that facilitate contact between the electrolyte ions and the MoO_x_ flakes. Moreover, the MoO_x_ flakes had a layered structure with many surface and edge defects, which provided abundant storage sites for anchoring extraneous ions during the electrochemical reaction process and promoted charge transfer. The unique MoO_x_ flake–based electrode displayed high electrochemical performance. This supercapacitor was shown to have a high areal capacitance of 112.5 mF cm^−2^ at 1 mA cm^−2^ and excellent energy density (15.64 µWh cm^−2^)/high power density (2.53 mW cm^−2^). The life span of supercapacitor implants can vary from a few days to a few weeks. The supercapacitor can be degraded through a series of metabolic and hydrolytic reactions, and the resulting byproducts can be absorbed by the body without any adverse long‐term effects, as they have been shown in rats.

A recent study reported the development of self‐powered wireless bioresorbable implants for programmed drug delivery that have a drug reservoir that acts as a battery to provide the power needed to open the gates of the device.^[^ ^288^ ^]^ The device was built with an electrochemically active metal foil that serves as an anode and an electropositive metal foil that serves as a cathode (Figure 7c). The two foils are interconnected by a conductive paste. Another connection joins the cathode to the collector terminals of phototransistors. When a phototransistor is exposed to external light, it accelerates the corrosion of the anode to open the underlying reservoir and release the drug. The implantation forms a battery with a collection of electrochemical cells, where biofluid serves as a common electrolyte. The drug reservoir itself acts as a battery to self‐power the implant and provide sufficient power needed to open the gate.

##### Studies—In Vitro Studies

In an in vitro study using osteoblast progenitor cells, a self‐powered stimulator made from a TENG and interdigitated electrodes of PET and PDMS was shown to promote cell adhesion, proliferation and differentiation^[^ ^284^ ^]^. Cell attachment and spreading started immediately after seeding. After three hours of stimulation, the number of attached progenitor cells increased by 72.76%, and the total spreading area increased by 78.37% after one hour of stimulation. Furthermore, cell proliferation was enhanced by 23.82% after three days of stimulation, and differentiation was increased by 28.2% after 12 days of stimulation (Figure 7d). Bacterial adhesion was effectively inhibited, the number of bacteria was reduced, and the live/dead bacteria ratio for the formation of mature biofilms was reduced by the use of a self‐powered anodized titanium implant with a TENG that was built to provide a long‐term effective negatively charged implant surface.^[^ ^289^ ^]^ The negatively charged implant surface also promoted the spread and differentiation of osteoblast progenitor cells without adverse side effects.

In vitro studies on self‐powered wound dressings were assessed against *E. coli* and *S. aureus* through a standard plate counting method and live or dead bacterial assays.^[^ ^269^ ^]^ The results indicated a temperature gradient‐dependent inhibition of bacterial growth. When the temperature gradient was increased from 0° to 15°C, the survival rate of *E. coli* decreased from 100 to 16%. The wound dressing was further examined to determine the correlation between the amount of H_2_O_2_ generated and the antibacterial performance. When the temperature difference was 15 °C, the functionalized Bi_2_Te_3_ NPs generated ≈4.4 µm H_2_O_2,_ which decreased the survival rates of *E. coli* and *S. aureus* to 12.7 and 18.7, respectively, within 30 min.

##### Studies—In Vivo Studies

In vivo studies conducted on adult mongrels (dogs) revealed that the I‐TENG behaved independently and charged capacitors on the basis of physical movements.^[^ ^285^ ^]^ For example, when an animal is active, the capacitor voltage rapidly increases, and when it is calm, the system load consumes energy when the storage voltage becomes saturated or decreases. The I‐TENG charged capacitors even from small movements when the animal was asleep. It is considered appropriate for body‐implantable medical materials. In another study, a flexible triboelectric active sensor (I‐TEAS) was implanted in pigs to monitor physiological and pathological signs by sensing the biomechanical motion of intracorporeal organs.^[^ ^286^ ^]^ In response to the heartbeat and breathing, an electric output with a short‐circuit current of ≈4 µA was generated, exhibiting the self‐powering capability of the system without the need for a battery. Moreover, the integration of nanostructured triboelectric layers and encapsulated materials enabled I‐TEAS to detect tiny changes in the motions of surrounding organs. The rates of heartbeats and respiration were precisely monitored, with no reduction in sensor properties. In another study, PENG was implanted on the surface of the heart of a rat and powered by stretching, which was repeatedly released as the heart expanded and contracted. The results indicate that the output signals were almost synchronized with the heartbeats.^[^ ^281^ ^]^

PENGs are implanted on the surface of the heart of a rat and are powered by stretch‐relaxation repeatedly as the heart expands and contracts. The results indicate that the output signals were almost synchronized with the heartbeats.^[^ ^281^ ^]^ When the rigid PENG was fixed at the apex of the pericardial sac, where the motion amplitude of the heart is larger than that at other sites, the maximal output open‐circuit voltage (V_oc_) and short‐circuit current (I_sc_) of 20 V and 15 µA, respectively, were obtained.^[^ ^282^ ^]^ The PENG‐powered pacemaker produced a pacing pulse with an amplitude of ∼2.5 V and a pulse width of ∼2 ms at a preset rate of 80 beats per minute, which was identical to the output of the pacemaker powered by an external battery. The heart can be continuously paced by a PENG‐powered modern full‐function pacemaker. In another study, after implantation of the PENG on the lateral wall of the left ventricle of dogs, PENG implantation did not burden the heart. It produces a voltage that is synchronous with the heartbeat. The PENG harvested as much as 0.487 µJ of electrical energy from every heartbeat, which is greater than the commercial pacemaker pacing threshold. The in vivo energy harvested by the PENG has powered a commercial pacemaker, generating pacing pulses at the preset parameters.^[^ ^283^ ^]^

In vivo degradation evaluation of an absorbable supercapacitor implant was performed in a rat.^[^ ^287^ ^]^ The implant was fully resorbed by the rat through metabolism after one month, with leakage of MoOx flakes and electrolyte from the broken implant, which was observed after three months, and complete dissolution was observed after six months. No inflammatory reaction was observed during the degradation process. In a recent study, a wearable self‐powered wound dressing was fabricated by integrating a TENG with the thermocatalyst bismuth telluride (Bi_2_Te_3_), which can be triggered by diverse stimuli (mechanical motion and a temperature gradient).^[^ ^290^ ^]^ When dressings are applied to infected wounds, the thermocatalyst produces H_2_O_2_ for in situ bacterial eradication, whereas the TENG generates electrical stimulation to promote wound healing. In vivo studies conducted on mice revealed that in the presence of an electric field (EF) generated by the TENG, fibroblast proliferation and migration were enhanced by ∼2.1‐ and ∼3.2‐fold, respectively, in wearable self‐powered dressings.^[^ ^269^ ^]^ After twelve days, the TENG‐treated wounds had completely recovered (<11%), while the relative wound closure rate of the control was still ∼31% (Figure 7e). The pulsed DC output from the TENG directed the cells toward the center from the edges, resulting in a progressive reduction in the wound area to 71.59% along the direction of the electric field.

In vivo studies of self‐powered electrical simulators were conducted by implanting stimulators on the surface of the femurs of rats (Figure 7f).^[^ ^284^ ^]^ The output measurements were conducted by pulling the legs of the rats with a linear motor, and the mechanical energy generated by leg movement was converted to electrical energy by the TENG. The energy was stored in the capacitor to drive the stimulator periodically (Figure 7f). The TENG identified the short‐circuit current, and the transferred charge reached approximately 1 nA. Studies have also been conducted in rats to study self‐powered devices that trigger drug release via light‐controlled mechanisms.^[^ ^288^ ^]^ A drug reservoir containing lidocaine was placed adjacent to the injured nerve. When the light passed, the drug was released from the reservoir, which induced a decrease in the grip strength and paw withdrawal threshold of the right hind limb, confirming the physiological effect of the release of lidocaine from the device. Although the work highlighted the biocompatibility and bioresorbability of the device, the use of nonresorbable phototransistors, optical filters, the amount of drug and number of reservoirs that can be equipped with implants, and the wavelength that can trigger release are challenges that still need to be addressed.

##### Studies—Clinical Studies

To date, no clinical studies have been reported.

##### Summary

Advances in current self‐powered implants have enabled the development of diagnostic and therapeutic BIMEs that allow in vivo sensing and tissue stimulation over long periods of time, which is relevant to clinical standards. Flexible configurations of self‐powered systems rely on the conversion of endogenous energy in the body, such as motion, flow, heat, or chemical changes. Advances in biodegradable implantable medical electronics (BIMEs), such as pacemakers and neurostimulators, have been achieved over the past few years due to advancements in materials development. Nonetheless, one of the standing challenges is the degradation of these self‐powered implants in the body. The degradation rates of these implants are not well controlled; they lack mechanical strength and induce inflammatory reactions in acidic media. Another challenge is to predict the exact degradation process in vivo, as it is influenced by the implant design and its location in the tissues. Biodegradable metals based on Mg, iron (Fe) and zinc (Zn) have been extensively studied for the development of biodegradable metallic implants. There is also a need to develop self‐powering implants with hybrid energy harvesters or systems that are capable of scavenging energy from multiple sources simultaneously, thereby counterbalancing any limitations caused by single units. Efforts should be focused on enhancing the lifetime of self‐powered implants. Although current self‐powered implants have achieved significant progress in integrating with biological systems, they are still in their infancy. The challenges include the lifespan of the system, degradability, how to achieve long‐term coupling and biosafety to comply with normal organ function, how to achieve volumetric energy density, and how to integrate effectively with other functional components. Multidisciplinary collaboration among scientists, engineers, and clinicians is needed. In addition, more large‐animal studies should be performed to enable clinical applications at a later stage.

#### 2.2.7 Self‐Oxygenation

**Figure 8:**
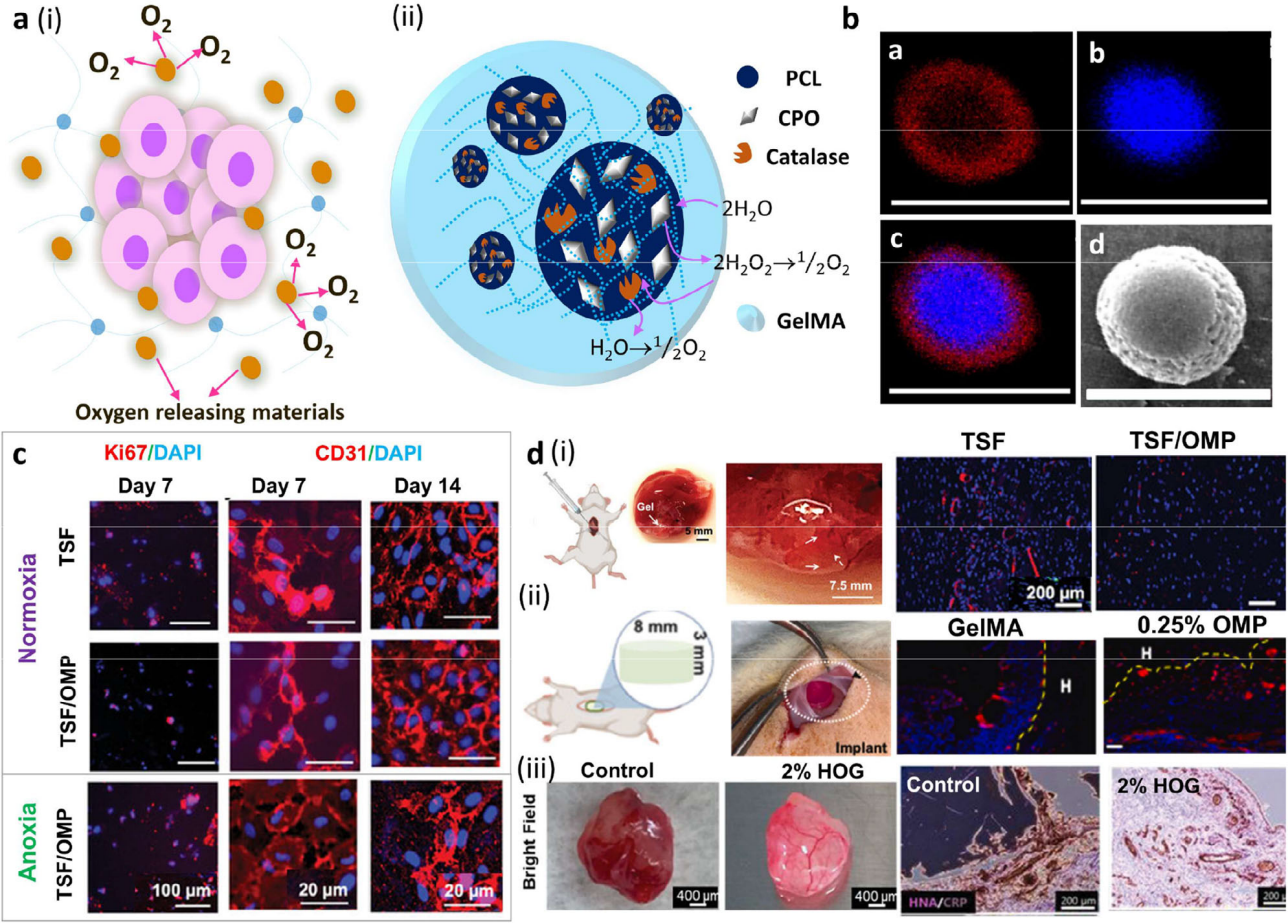
Self‐oxygenating implants. Concept of self‐oxygenating implants. a, i) Illustration showing self‐oxygenating materials and the release of oxygen (O_2_) from the GelMA nanocomposite hydrogel. Mechanisms. ii) Schematic illustration of the reactions underlying O_2_ generation via CPO hydrolysis and H_2_O_2_ decomposition by catalase in hydrophobic O_2_, generating microparticles encapsulated with CPO. b) Material studies: Core‐shell structure of O_2_‐releasing microspheres and O_2_ release kinetics: a) PLGA shell, b) PVA/H_2_O_2_ core, c) combined core‐shell structure, and d) SEM image of microspheres.^[306]^ c) Cell studies: studies on HUVECs showing enhanced cell survival with the use of Ki67 staining and CD31 expression on TSF gel in normoxic and anoxic conditions after 14 days of culture. d) In vivo studies: i) TSF‐based therapeutic hydrogel implanted at the infarct area. Representative image of HIF‐1α staining in the left ventricular ischemic region 3 weeks post‐injection.^[^ ^298^ ^]^ ii) Microphotographs of histological sections of GelMA and 0.25% OMP stained for neovascularization (CD31) of the hydrogels revealed slow degradation and less neovascularization in the GelMA constructs even at 3 weeks post‐implantation.^[^ ^322^ ^]^ iii) Bright‐field images of explants taken seven days post‐implantation. Micrographs of 5‐micrometer‐thick midsagittal sections of implants that were stained with HNA/CRP. Semiquantitative image analysis of the core and outside of the HNA‐stained tissue sections revealed that hMSCs of self‐oxygenated tissues remained present in the implant and were located around the vessels.^[^ ^317^ ^]^ b) Reproduced with permission.^[^ ^305^ ^]^ Copyright 2018, Nature∖; c, d‐i) Reproduced with permission.^[^ ^298^ ^]^ Copyright 2024, Wiley‐VCH; d, ii) Reproduced with permission.^[^ ^322^ ^]^ Copyright 2023, Elsevier; and d, iii) Reproduced with permission.^[^ ^317^ ^]^ Copyright 2021, Wiley‐VCH.

A self‐oxygenated implant is an implant that can generate O_2_ via the incorporation of O_2_‐generating materials.^[^ ^292^, ^293^, ^294^ ^]^ Cells require a continuous supply of O_2_ to survive. Keeping cellular implants alive is a challenging task because angiogenesis takes a week or two to form vessels that can supply these implants after their implantation in the body, a time that is too long for cells to wait. Therefore, the development of self‐oxygenated implants is appealing. The concept of self‐oxygenated implants or tissue constructs is new and is based on self‐oxygenated materials that can potentially store and supply O_2_ on demand (**Figure** **8**). Moreover, O_2_ gradients can potentially guide stem cell fate toward osteogenesis.^[^ ^295^ ^]^ Materials such as self‐oxygenated bioinks for 3D bioprinting have been developed to fabricate tissue constructs^[^ ^296^, ^297^ ^]^ and injectable oxygenated hydrogels for cardioprotective procedures.^[^ ^298^ ^]^

The tissue engineering approach employs mammalian cells to regenerate the structure. However, cells mostly die after transplantation because of the lack of blood supply.^[^ ^299^ ^]^ Approaches that employ vascularized tissues, microvascular anastomosis, angiogenic growth factors, and endothelial cells have shown limited success.^[^ ^300^, ^301^ ^]^ Recently, the generation of O_2_ from materials included in the constructs has been explored.^[^ ^297^, ^302^ ^]^ It aims to provide cells with O_2_ while angiogenesis is taking place, with the hope of having an impact on the outcome of cellular implants in the body (Figure 3a‐iv).

##### Mechanism

Self‐oxygenation can be achieved by incorporating O_2_‐generating materials such as calcium peroxide (CPO),^[^ ^303^ ^]^ hydrogen peroxide (H_2_O_2_),^[^ ^304^, ^305^ ^]^ perfluorocarbon compounds,^[^ ^306^, ^307^ ^]^ magnesium peroxide (MgO_2_)^[^ ^308^ ^]^ and sodium percarbonate (Na_2_CO_3_),^[^ ^309^, ^310^, ^311^ ^]^ which can store and supply O_2_ on demand. CPO is the most commonly used material and generates O_2_ via the intermediate formation of hydrogen peroxide, as presented in the following equation:^[^ ^312^ ^]^

(1) $$\mathrm{Ca}O_{2}+2H_{2}O\to \mathrm{Ca}{\left(\mathrm{OH}\right)}_{2}+H_{2}O_{2}$$

(2) $$2H_{2}O_{2}\to 2H_{2}O+O_{2}$$

A lower concentration of hydrogen peroxide can lead to the formation of free radicals that can lead to cell death.^[^ ^313^, ^314^ ^]^ To protect against cell damage, various antioxidants and scavenging enzymes, such as catalase, superoxide dismutase and glutathione peroxidase, are used to hydrolyze peroxides into water and O_2_.^[^ ^315^, ^316^ ^]^ Recently, hydrophobic O_2_‐generators (HOGs), which can readily integrate within engineered tissues, have been developed (Figure 8a).^[^ ^317^ ^]^ There are concerns that oxygenation may inhibit angiogenesis, which is stimulated by hypoxia‐inducing factors. However, in a study in which O_2_‐generating particles were delivered to the heart of mice, endothelial cells were enhanced, and improved vascularization led to an increase in cardiomyocyte survival of ≈30%.^[^ ^298^ ^]^ Thus, regulating O_2_ levels within implants is important not only for cell metabolism but also for the development of blood vessels and ECM components.^[^ ^318^ ^]^

##### Studies—Concept studies

Studies have demonstrated the possibility of locally generating O_2_ by using peroxides (liquid and solid),^[^ ^319^, ^320^ ^]^ or cyanobacteria.^[^ ^321^ ^]^

##### Studies—Material Studies

The oxygenating ability of injectable tyramine‐conjugated alginate and silk fibroin encapsulated with oxygen‐releasing microparticle (TSF/OMP) hydrogels was tested via O_2_ release experiments by suspending the gels in culture media and saline and measuring O_2_ release for 14 days.^[^ ^298^ ^]^ The incorporation of OMPs into hydrogels significantly altered O_2_ release kinetics. The composite hydrogels displayed steady O_2_ release for two weeks, followed by a decrease of 3–4% by the sixth day. This decrease was due to the entrapment of the released gaseous O_2_ in the hydrogel network, which was not detected by the O_2_ sensor probe. TSF/OMP hydrogels maintained a steady O_2_ level of <5%, which is thought to be suitable for alleviating anoxic injury.

In a recent study, a self‐oxygenated injectable hydrogel composed of tyramine (TA), alginate and stromal cell‐derived factor‐1 alpha (SDF) and encapsulated O_2_‐releasing microparticles (OMPs) was used for the sustained release of SDF and O_2_ (Figure 8b).^[^ ^298^ ^]^ Alg was conjugated by TA via chemical crosslinking with tyrosine in silk fibroin (SF) to obtain hybrid TA‐conjugated Alg and SF (henceforth referred to as TSF) hydrogels. SF was introduced to improve the elasticity and toughness of alginate hydrogels. To achieve injectability, enzymatic crosslinking was performed via the use of horseradish peroxidase (HRP) and hydrogen peroxide to crosslink TA‐conjugated Alg, SF and SDF in situ after injection. This helps chemically crosslink TA‐Alg and SF hydrogels to tissues and aromatic amino groups of proteins to the ECM. In another approach, OMPs were incorporated into GelMA containing silica nanoparticles under different O_2_‐deficient conditions.^[^ ^322^ ^]^ In this study, amphiphilic GelMA chains were coated on the surface of hydrophobic OMPs, while the hydrophilic amino acids of the GelMA chains interacted with the water phase to produce a homogeneous dispersion of OMPs in the GelMA hydrogels. CPO‐incorporated hydrophobic PCL microparticles are able to generate O_2_ and can be used as self‐oxygenating implants (Figure 8c,d).^[^ ^317^ ^]^

##### Studies—In Vitro Studies

The effects of the injectable TSF/OMF hydrogels on the survival, proliferation, and endothelialization of HUVECs in ∼0.5% O_2_ (anoxic) and ∼5% O_2_ (normoxic) environments were assessed.^[^ ^298^ ^]^ The results from a 14‐day study revealed that the hydrogels maintained > 90% cell viability under both conditions. Furthermore, the metabolic activity after 14 days of culture was greater for Ki‐67‐producing hydrogels than for pristine hydrogels cultured under both anoxic and normoxic conditions. It was concluded that the TSF/OMP hydrogel induced increased endothelial cell proliferation.

**Figure 9:**
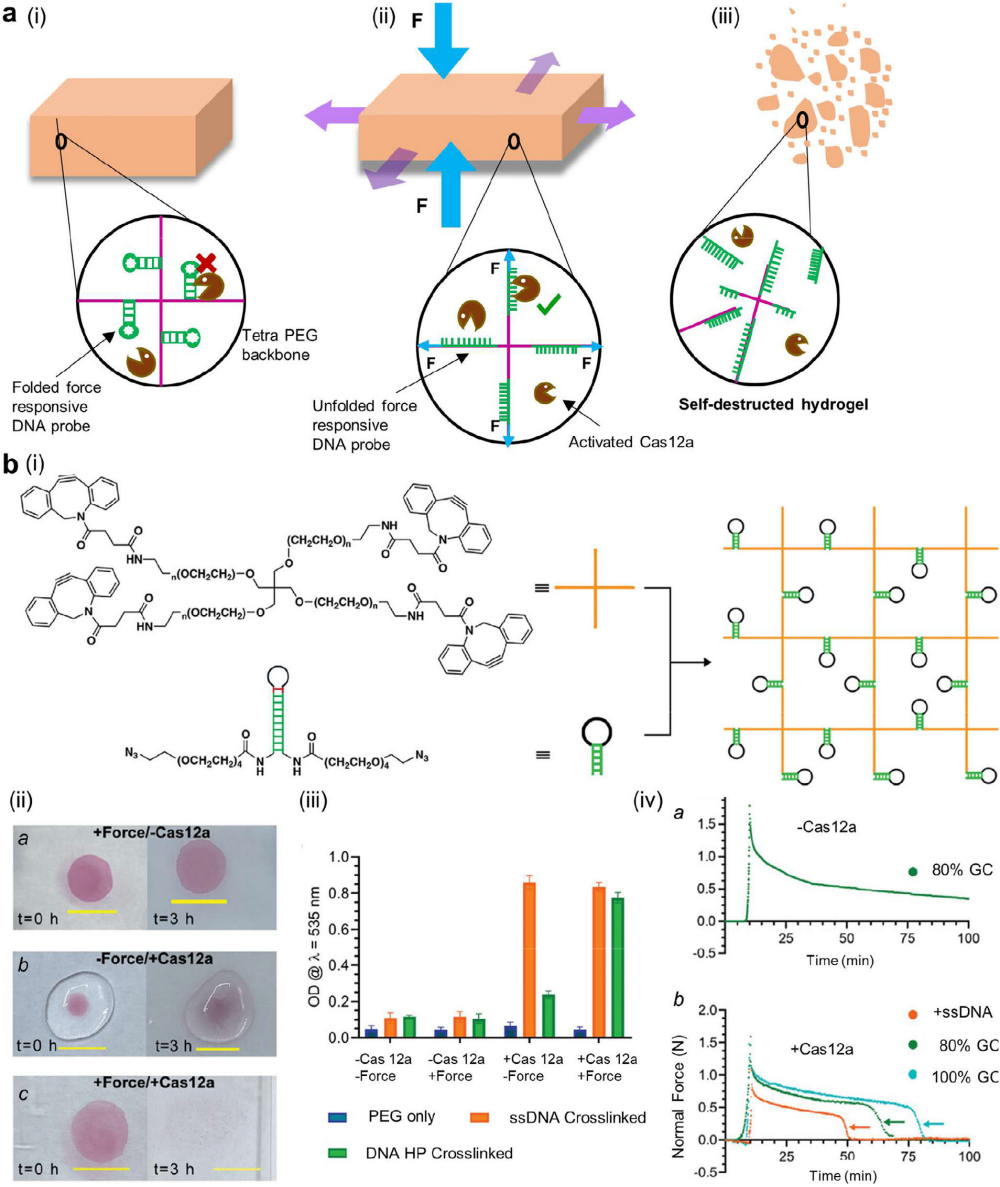
a) Illustration of the mechanism of self‐destructive hydrogels. i) Hydrogel consisting of DNA crosslinks and tetra‐PEG. ii) Force‐induced opening of DNA HP sites, which leads to CRISPR–Cas12a cleavage of the DNA. The black and blue arrows represent the external forces and the forces of the polymer backbone, respectively, and iii) self‐destruction of the hydrogel due to the cleavage of ssDNA crosslinks. b) Material studies: i) Synthesis of a self‐destructive hydrogel by crosslinking tPEG DBCO and bisazide‐modified DNA. ii) Images of hydrogels under different conditions. *a*) Hydrogel prepared without Cas12a under external force. *b*) Cas12a without an external force and *c*) Cas12 with continuous external force application. The photographs were taken at *t* = 0 and 3 h of continuous external force. iii) Graph showing the degradation of crosslinked hydrogels by tPEG‐N_3_, ssDNA and 80% GC HP. iv) Plots showing the stress relaxation of hydrogels without the Cas12a enzyme (*a*) and with the Cas12a enzyme (*b*). In the presence of the Cas12a enzyme, the stress suddenly decreased depending on the GC content of the HP. The ssDNA crosslinked hydrogel degraded at ∼50 min, and the DNA HP degraded at 65 min. The hydrogels with 100% GC HP were the most resistant to Cas12a activity and degraded at approximately 80 min. a–e) Reproduced with permission.^[^ ^348^ ^]^ Copyright 2023, Wiley‐VCH.

Moreover, O_2_ gradients can potentially guide stem cell fate toward osteogenesis.^[^ ^295^ ^]^ In vitro bulk mRNA sequence analysis of the nanocomposite hydrogels containing OMP at different concentrations was performed.^[^ ^322^ ^]^ hMSCs were encapsulated in GelMA hydrogels containing OMP (0.25%). The hydrogels were cultured under normoxia/anoxia in osteogenic differentiation medium for 28 days. A global analysis revealed that gene expression under anoxic conditions increased toward osteogenesis in the OMP group at 14 and 28 days, indicating that modulation of local O_2_ tension via self‐oxygenating hydrogels can potentially govern the differentiation of hMSCs toward osteogenesis in engineered tissue constructs/implants for bone defects (**Figure** **9**e). In another study, hMSCs encapsulated in GelMA/OMP hydrogels presented a higher rate of cell survival and proliferation than did those encapsulated with GelMA/silica nanoparticles (SNPs) at two weeks.^[^ ^322^ ^]^ Analysis of gene expression for osteogenesis revealed that, under anoxic conditions, the expression of genes in the OMP constructs, such as bone morphogenetic protein 6 (BMP6), activating transcription factor 4 (ATF4), the catenin beta 1 encoding gene (CTNNB1), hairy and enhancer of split‐1 (HES1) and DExD‐Box helicase 1 (DDX21), was upregulated, indicating the promotion of osteogenesis by self‐oxygenated hydrogels.

In one study, a GelMA hydrogel incorporated with calcium peroxide improved the function of human cardiac cells cultured under hypoxic conditions.^[^ ^323^ ^]^ The viability of OMPs incorporated into GelMA hydrogels containing silica nanoparticles decreased from approximately 90% to less than 80% under normoxia (human mesenchymal stem cells (hMSCs)).^[^ ^322^ ^]^ In vitro studies have investigated the ability of HOGs to protect cells from the cytotoxic effects of generated hydrogen peroxide.^[^ ^317^ ^]^ Self‐oxygenated tissues were produced by incorporating hMSCs in GelMA hydrogels containing different concentrations of CPO or HOGs. Under normoxic conditions, the addition of CPO led to massive cell death even at low concentrations (2.5%), whereas self‐oxygenated tissues generated from HOGs presented a minor decrease in cell survival at high concentrations (10%), which confirmed the cytoprotective nature of these materials. Under anoxic culture conditions, these self‐oxygenated tissues enriched with HOG‐GelMA formulations enabled ∼80% cell viability for 12 days, even at low concentrations (2.5%). The cell survival rates were identical under both conditions, which indicated that HOGs effectively prevented cell death throughout the volume of self‐oxygenated tissues. O_2_ generated by cyanobacteria,^[^ ^321^ ^]^ was shown to eradicate biofilm formation by *Streptococcus gordonii*, *P. gingivalis, Fusobacterium nucleatum and Porphyromonas gingivalis*. Similarly, self‐supplying H_2_O_2_‐modified nanozyme‐loaded hydrogels were shown to prevent biofilm formation by *Enterococcus faecalis* and *Streptococcus sanguis*.^[^ ^324^ ^]^

##### Studies—In Vivo Studies

Studies on rat acute myocardial infarction (MI) have shown that TSF/OMP hydrogels lead to a reduction in fibrotic areas.^[^ ^298^ ^]^ Furthermore, they induced more myocardial vessel formation than did the pristine hydrogel. The protein begins to degrade at two to three weeks post‐implantation. Although the expression of HIF‐1α, which encodes a gene important for angiogenesis, was found to decrease in animals treated with the TSF/OMP hydrogel, decreased cell death was observed due to oxygenation (Figure 8d‐i). O_2_ is also crucial for maintaining healthy endothelia and achieving neovascularization. Qualitative analysis revealed that angiogenesis was promoted and that vascular networks were more organized in hearts treated with the TSF/OMP or TSF/OMP/SDF hydrogels. In another study, self‐oxygenated (GelMA/OMP) hydrogels implanted on the backs of rats were found to undergo slow biodegradation, resulting in increased cell proliferation, CD31+ cell vascularization, and vessel diameter (Figure 8d‐ii).^[^ ^322^ ^]^ The use of self‐oxygenated injectable hydrogels that encapsulate O_2_‐releasing microparticles and are used for the sustained release of SDF and O_2_ at MI sites in rats was found to prevent hypoxia‐induced cell death better than the use of OMPs or SDF alone.^[^ ^298^ ^]^ Although studies have investigated the effects of O_2_‐generating materials on the functional behavior of implants, such as hypoxia,^[^ ^325^, ^326^ ^]^ osteochondral bone defects and other disease conditions,^[^ ^327^, ^328^ ^]^ their actual function inside the body has not yet been investigated. Engineering tissue implants with complex geometries of vascular networks and achieving complete integration with host tissues are major challenges since they not only enable the delivery of O_2_ but also increase the nutrient supply and help in the removal of metabolic waste.

In vivo experiments in rats revealed that HOG‐mediated self‐oxygenated tissues composed of GelMA hydrogels containing 2 × 10^6^ cells, hMSCs and HOGs presented a healthy pink appearance with complex patterns resembling a vascular structure, whereas the control group presented a dark color (Figure 8d‐iii)^[^ ^317^ ^]^ seven days post‐implantation. Human nuclear antigen (HNA) staining revealed that the hMSCs of self‐oxygenated implants remained in situ, predominantly around vessels (Figure 8d‐iii). In addition, engineered tissues also contribute to integration with host tissues (human nuclear antigen (HNA) and CD31+), suggesting the growth of the host vascular system.

In another study, _an_ O2‐releasing BCP scaffold incorporated with CPO was implanted in rabbits to enhance bone repair in vivo. Compared with that of the bare scaffolds, bone regeneration was significantly greater in the CPO‐coated scaffolds. Six months post‐surgery, new bone formation was evident around the periphery of the scaffolds and along the native bone.^[^ ^329^ ^]^

##### Studies—Clinical

To our knowledge, no clinical studies exist.

##### Summary

An inadequate supply of O_2_ after implantation may lead to constraints on cells, including inadequate angiogenesis, leading to improper function of the implant. Advanced technologies such as 3D printing and microfluidic chips may help us improve implants by controlling and providing a uniform O_2_ supply in future implants. HOGs are promising approaches for developing clinically sized tissues for regenerative applications.^[^ ^317^ ^]^ Self‐oxygenated implants are evolving as a new type of implant that can be designed into tissue constructs, complex vessel networks, hydrogels, or scaffolds that can possibly solve O_2_ deficiencies. In the future, there is also a need to integrate self‐regulating components into engineered implants for a controlled on‐demand supply of O_2_. Moreover, the use of self‐oxygenating materials that can be triggered by changes in local pH can be useful for implants. Self‐oxygenated devices are furturistic designs of autonomous implants and are expected to advance in the coming years. Currently, available tissue constructs have many limitations, such as management of the O_2_ supply to the target cells/tissues, proper O_2_‐generating sources, assembly with an implant, and proper degradation, which require further research and testing through large animal models to obtain a substantial understanding that can help us take the next steps for clinical applications. The integration of sensor technology with implants could help monitor internal conditions. Regulatory approval of self‐oxygenating implants is another challenge, and approval and commercialization may take longer than other implants.

#### 2.2.8 Self‐Destruction

A self‐destructive implant is an implant with the ability to degrade autonomously when exposed to triggers. Self‐destructive biomaterials can degrade upon exposure to external stimuli such as chemical cues^[^ ^330^, ^331^, ^332^ ^]^ and optical excitations,^[^ ^333^ ^]^ which initiate a self‐disintegration process that drives the cleavage of labile linkages proximal to the core polymer networks in the biomaterial. These materials can be helpful in controlled release applications, where their degradation leads to the release of prodrugs^[^ ^334^ ^]^ and enzymes^[^ ^335^, ^336^ ^]^ entrapped inside the biomaterial network.

Although many of these autonomous implants are biostable, biodegradable implants can also be autonomous. This includes biodegradable sensing components, which constitute an advancing frontier in science. Biodegradable electronic devices are emerging as innovative therapeutic platforms, offering advantages such as the avoidance of removal‐associated risks, reduced risk of infection, and avoidance of the cost of a second procedure, as well as patient anxiety and stress associated with an additional procedure for implant removal. These devices are made from materials that enable reliable functionality over a clinically relevant timeframe before they safely degrade into nontoxic byproducts.^[^ ^337^, ^338^ ^]^ For example, a fully bioresorbable, implantable, cardiac pacemaker that operates in a battery‐free manner in mice has been shown to be an effective treatment for atrioventricular (AV) nodal heart block.^[^ ^338^ ^]^ Tests in a canine model suggested potential suitability for adult human applications.

Another example is the use of bioresorbable electrical stimulators that hold significant promise, offering temporary functionality followed by complete and safe dissolution. A key application is the treatment of peripheral nerve injuries through targeted electrical stimulation at proximal or distal sites, which is traditionally effective for durations of up to one week.^[^ ^339^ ^]^ Recently, an advanced class of bioresorbable stimulators that extend both functionality and therapeutic impact was reported.^[^ ^339^ ^]^ These enhanced devices support 1) simultaneous stimulation at both proximal and distal nerve sites and 2) long‐term operation lasting several months, all while being fully bioresorbable via hydrolysis in biofluids.^[^ ^339^, ^340^, ^341^, ^342^ ^]^ In vivo studies have demonstrated the efficacy of stimulators in promoting peripheral nerve regeneration, as evidenced by increases in total muscle mass, muscle fiber cross‐sectional area, and compound muscle action potentials.^[^ ^339^ ^]^ These results underscore the potential of next‐generation bioresorbable stimulators not only for short‐term therapy but also for extended neuromodulation and regenerative applications in clinical settings.^[^ ^339^ ^]^

Despite their advantages, biodegradable electronic devices are associated with certain challenges, including biocompatibility. Addressing this challenge will pave the way for seamless integration of bioresorbable electronics into patient care.^[^ ^343^, ^344^, ^345^ ^]^ Another challenge is signal stability due to biofouling. For example, enhancing the signal stability of implantable biosensors requires effective management of biofouling and the body's foreign object response.^[^ ^346^ ^]^ Extensive research has explored both passive and active strategies to address these challenges. Passive approaches rely on advanced material designs, such as hydrophilic, biomimetic, drug‐releasing and zwitterionic polymers that inherently resist protein adsorption and cell adhesion. Active methods, on the other hand, employ stimulus‐responsive materials and integrated electromechanical or electromagnetic transducers to prevent or reverse biofouling dynamically. Together, these strategies contribute to maintaining sensor performance and reliability in long‐term biomedical applications.^[^ ^347^ ^]^ Challenges also include manufacturing costs. The production of transient biosensors for implantable applications is costly, limiting their wide clinical adoption. This challenge is largely driven by the expense of specialized materials and intricate fabrication techniques, particularly for custom or miniaturized devices. To address this, researchers are pursuing multiple cost‐reduction strategies. One approach focuses on the use of low‐cost, widely available materials such as conventional polymers and metals.^[^ ^347^ ^]^ Another approach emphasizes the development of scalable, high‐throughput manufacturing methods. One reliable method discussed in this review is the use of 4D printing.

##### Mechanism

Self‐destruction can be achieved by force triggering a polymer using a DNA‐linked hydrogel. In a recent study, DNA hairpins were used as force sensors for structural transition, and an RNA‐guided endonuclease (CRISPR Cas 12a) was used as a selective degradation system to activate DNA hairpins under mechanical strain (Figure 9ai‐iii).^[^ ^348^ ^]^

##### Studies—Concept studies

To date, no studies have been conducted on self‐destructive materials based on this concept.

##### Studies—Material Studies

The self‐destruction of hydrogels was studied by introducing Cas12a into hydrogels synthesized from DNA hairpins (DNA HPs) with different GC contents and doped with gold nanoparticles (AuNPs).^[^ ^348^ ^]^ The hydrogel crosslinked with ssDNA and GC showed complete self‐destruction within 30 min under zero external force, indicating that the thermal breathing of DNA HPs contributed to the destruction of the hydrogels (Figure 9b‐i). When both Cas12a and external forces were applied, self‐destruction of the hydrogel occurred after three hours of incubation, and the release of AuNPs occurred. It was predicted that force‐induced self‐destruction occurred due to the opening of the DNA HP crosslinkers inside the hydrogel, which triggered Cas12a breakdown of the DNA crosslinker, ultimately leading to self‐destruction of the hydrogel. Rheological studies carried out on the material following the application of stress and the Cas12a enzyme showed that the decay of the self‐destructive material was initially slow, but it suddenly decreased to zero after a certain period. It was predicted that initial relaxation was caused by hydrogel relaxation behavior, whereas the sudden drop in stress was due to self‐destruction of the gel (Figure 9b‐ii‐iv).

##### Studies—In Vitro Studies

No studies on this topic have been reported thus far.

##### Studies—In Vivo Studies

No studies on this topic have been reported thus far.

##### Studies—Clinical Studies

No studies on this topic have been reported thus far.

##### Summary

Self‐destruction is an important concept activated upon exposure to triggering stimuli, which can be useful in applications such as the release of lead. The application of external forces triggers degradation, e.g., DNA crosslinking and cleavage of the network in the hydrogel, and, finally, self‐destruction. Self‐destructive materials can also be used for sensing and other applications. To date, in vitro studies have not been reported. There are no preclinical animal or clinical studies. Self‐destructive materials are expected to soon have a place similar to that of other stimuli‐responsive materials in clinical applications, such as in the treatment of infection, cancer, and inflammatory diseases.

## 3 Assembly of Different Properties into the Target Implant

The evolution of concepts toward the realization of autonomous implants started with the development of implants that have only one aspect of autonomy, e.g., self‐healing. This was then followed by the integration of two functionalities, e.g., self‐sensing and self‐powering, leading to the development of the second generation of autonomous implants. The third generation includes three or more properties. Ultimately, with a number of integrated functionalities (4th generation), the ideal implant should closely mimic native tissues to be able to sense, actuate, heal, remodel and interact successfully. To dissect these developmental steps and help analyze achievements and visions, we classify these implants into these three generations and discuss aspects related to each of them in this section. The aspects discussed include each generation definition, aim, fabrication, characterization, scale‐up, clinical application, regulation and current status with their development.

### 3.1 First Generation

The first generation of automated implants can be defined as implants with one specific property, e.g., self–healing, self‐sensing, self‐actuation or self‐powering properties. The aim of using the first generation of autonomous implants is to advance technology beyond the current state‐of‐the‐art of implants used in the clinic, i.e., having some smart property that enables, e.g., self‐awareness, self‐healing, sensing, actuation or communication. Examples include in vitro and in vivo experiments involving the development of self‐healing implants for the treatment of brain injury,^[^ ^118^, ^349^ ^]^ Parkinson's disease, self‐healing hydrogels for the treatment of diabetic wounds,^[^ ^350^, ^351^ ^]^ self‐powered knee implants^[^ ^352^ ^]^ and cardiac pacemakers,^[^ ^353^ ^]^ self‐sensing implants for collecting data on bone healing and monitoring the bone‒implant interface.^[^ ^22^, ^354^ ^]^

For the fabrication of the first generation of autonomous implants, a) appropriate biocompatible materials and b) appropriate designs, which should consider all the biomechanical and biological factors that are required for proper function, e.g., a self‐aware implant spine cage requires high‐resolution imaging and computer modeling for its design, which enables matching of the bone structure and joint mechanics. Other techniques, such as 3D computer‐aided design (CAD) models,^[^ ^355^, ^356^ ^]^ help in the fabrication of intricate structures with restricted geometries. Various types of nanosized structures, such as metal‒organic frameworks, nanorods, nanocubes, quantum dots and nanopillars, that are used in the fabrication of implants are very important to consider.^[^ ^357^, ^358^ ^]^ c) Creating complex geometries. Techniques such as 3D printing allow the creation of customized implants that can fit individual patient anatomy and needs. e) Fabrication protocols need to be developed that can be used for commercial purposes, e.g., the fabrication of self‐healing hydrogels can be improved by developing new theories on self‐healing mechanisms.

For the testing of autonomous implants, i.e., self‐aware implants, mechanical tests should be performed to evaluate the static and dynamic fatigue properties via standard protocols such as the American Society of Testing and Materials (ASTM) and different spine models. The mechanical properties must be tuned on the basis of the requirements. The nanogenerators used in self‐powered implants need to be monitored, and the lifetimes of these nanogenerators have to be studied in animal models. We need advanced techniques to study self‐aware implants when placed in an animal model. The application of artificial intelligence (AI) is useful for characterizing implants via complex algorithms to generate useful outputs.^[^ ^359^ ^] [^ ^360^ ^]^ To optimize biomechanical models, in vivo studies are needed: b) preclinical testing of new implants and c) monitoring of the process after the insertion of the implant. For example, for self‐healing implants, in situ evaluation through quantitative techniques and nondestructiveness are needed.^[^ ^361^ ^]^ Advanced morphological techniques and multiscale characterization are needed for a better understanding of atomic‐ and molecular‐scale observations, which can be converted into quantitative and numerical information.^[^ ^362^ ^]^ Advanced numerical healing kinetics could be used to design self‐healing materials that are suitable for self‐healing implants.^[^ ^363^ ^]^

Scale‐up will be needed to develop small‐scale energy generators for self‐aware and self‐powering implants with the ability to provide sufficient and continuous energy supply to the implants. Many analyses, such as technical and design considerations and manufacturing processes, must be carried out before scaling up and manufacturing first‐generation implants. Implant function and durability must be proven in short‐ and long‐term in vivo studies, including the use of specific models for target applications. In addition to functionality, the biocompatibility of the implant and byproducts must be demonstrated before moving to clinical studies. To date, no clinical studies on first‐generation autonomous implants have been reported. This is largely due to the lack of long‐term and comprehensive in vivo studies, which should be pursued in the course of developing this type of implant.

According to the EU Medical Device Regulations, orthopedic implants (e.g., self‐powered knee implants) belong to class II, the highest risk class.^[^ ^364^ ^]^ Currently, it is challenging for these autonomous implants to undergo the same Food and Drug Administration (FDA) or EU approvals, as the overall process takes years to complete and involves extensive clinical trials before approval is granted. Regulatory approval of the autonomous implant involves biocompatibility testing, which determines if the implant is safe while in the body (such as for ruptures, infections or complications), effective, manufacturing standards, etc.

Currently, the first generation of autonomous implants has been well developed and has been proven to work in many in vitro and in vivo studies. However, long‐term, large animal studies and specific application models are still lacking, e.g., for self‐healing,^[^ ^115^, ^118^ ^]^ self‐powered^[^ ^290^ ^]^ and self‐regenerating implants.^[^ ^162^ ^]^ These findings are needed to advance to clinical applications. Self‐powered implants (TENGs driven by low‐frequency mechanical sources) are under consideration for enhancing the dielectric constants of triboelectric materials. There is a growing focus on bioresorbable TENGs,^[^ ^365^, ^366^ ^]^ needle‐sized injectable TENGs^[^ ^367^ ^]^ and self‐powered wireless monitoring systems. On the other hand, no in vitro or in vivo studies have been reported on self‐aware implants. The experiments conducted thus far are still at the conceptual level, where advances in nanogenerators and metamaterials could play a crucial role and guide the development of next‐generation multifunctional implants that could be used by clinicians to achieve better surgical outcomes. Other types of first‐generation autonomous implants are being studied in vitro and in experimental animals.

Emerging research has demonstrated the possibility of using mechanical metamaterials to create engineered architectures whose effective properties arise from their geometry rather than their bulk chemistry.^[^ ^368^, ^369^ ^]^ Therefore, they can be developed to have tunable stiffness and anisotropic behavior,^[^ ^368^, ^370^ ^]^ making them useful for the development of autonomous implants. A pilot “electronic‐free” knee implant combining piezoelectric nanogenerators with a tailored metamaterial scaffold to harvest mechanical strain energy, self‐powered a wireless transmitter, and reported joint loading through tissue without an onboard battery was reported.^[^ ^371^ ^]^

Electromagnetic metamaterials can be used for attenuation and mismatch of RFC between biomedical implants associated with radio frequency release or reception inside the human body to contribute to the development of autonomous implants in the future. For example, such implants can receive external radio frequency communication to start or stop autonomous functions.

Self‐powered implants still require surgical removal for recharging energy storage, thereby imposing physical and psychological strain on patients. To improve the system, we need more self‐powered implants that can potentially self‐recharge or extend the operation time inside the body and reduce the risk of surgery, such as self‐rechargeable cardiac pacemakers^[^ ^285^ ^].^ Self‐powered implants integrated with wireless control of programmed drug delivery will be the next step,^[^ ^288^ ^]^ and they could provide solutions for long‐term powering and monitoring of the physiological condition of the patient. Therapeutic approaches for designing self‐healing implants for the treatment of brain diseases are advancing; however, further improvements are needed when these implants are translated into humans. The implants should mimic the mechanical properties of brain tissue while maintaining other functionalities, including adhesion, conductivity, and anti‐inflammatory, antioxidant and hemostatic properties. When implanted, cells or drugs should be able to effectively migrate and interact with brain tissues and initiate the healing process. The overall mechanism needs a deeper understanding.

### 3.2 Second Generation

The second generation of autonomous implants combines two autonomy characteristics, e.g., spinal fusion implants with self‐sensing and self‐powering features.^[^ ^16^ ^]^ Combining more than one self‐performing function will increase the effectiveness of the implant, reduce risks and increase the likelihood of realizing autonomous implants that can mimic native tissues. The fabrication of second‐generation implants requires a special design and sometimes different fabrication techniques to include two characteristics of one implant. For example, self‐aware and self‐powering implants require the use of meta‐tribomaterial technology with multiple layers of triboelectronic auxetic microstructures with multistable or self‐recovering snapping segments to achieve self‐awareness ^[^ ^17^ ^]^ and nanogenerators to achieve self‐powering. The system relies on the rational design of implants to embed advanced functionalities into the matrix. By manipulating the geometrical design, the implant can be transferred into a sensor or a nanogenerator. The integration of multiple circuit boards, powering sources into the implant, scalability and availability of biocompatible raw materials for the fabrication of these implants is another challenge.

This generation of implants needs to undergo specific testing to demonstrate that they have two functions, which do not interfere with each other. For example, self‐sensing and self‐powering‐based spinal fusion cage implants, cardiovascular stents and shock absorbers need to be tested in small and large animals before their use in clinical applications. The process, materials and design of the implants must be optimized by interconnecting various parameters that can create the basis for the scale‐up of 2^nd^‐generation implants. The mass production of materials is needed for designing implants. Recent advances in nanocomposite materials^[^ ^372^ ^]^ have led to the creation of a broad range of designs and technologies that allow engineers to identify appropriate materials for mass production. Although self–aware implantation via meta‐tribomaterial technology is a step forward, the development of robust and long–term performance evaluations of these implants in vivo is needed. Clinical trials need to be conducted using these implants before they can be practically brought into regular use.

Appropriate studies and detailed reports of studies on material properties, behavior, biocompatibility and durability are needed. In the European Union (EU), these implants and devices are regulated by the Active Implantable Medical Devices Directive (AIMDD), the Medical Devices Directive (MDD) and the In Vitro Diagnostic Medical Device Directive (IVDMDD).^[^ ^373^ ^]^ In the US, the FDA regulates the safety and effectiveness of implants. Federal regulations (such as the Code of Federal Regulations) must be fulfilled for the Center for Medical Devices and Radiological Health (CDRH) to approve implants in the U.S. Although technology is still at the concept level, as self‐aware orthopedic 2^nd^ generation implants have both diagnostic and energy harvesting capabilities, they have the potential to help in the assessment of the bone healing process around them.^[^ ^16^, ^374^ ^]^ The fabrication of actual models still needs to be performed, and in vitro and in vivo studies need to be carried out.

### 3.3 Third Generation

Third‐generation implants can be defined as implants formed by combining three or more autonomy characteristics. Multiple self‐functioning implants are integrated into one autonomous implant, which is the next step closer to native tissues. The third generation can be considered a futuristic implant with multiple features that is adaptable and tunable on the basis of specific needs. A combination of automation and flexibility in design is crucial. We need hybrid materials and technologies that combine natural and synthetic materials in appropriate ratios, and each material provides functionality. The assembly of individual self‐units, e.g., self‐powering, self‐actuating, and self‐healing implants into a single unit, requires technological development (including powering units, nanogenerators), material development (biomaterials, nanocomposites) and method development (2D, 3D, and 4D). The current need is to improve the fabrication method to a higher level of control and accuracy with automatic fabrication methods while retaining a clinically relevant production rate. Integrating wireless data logging technology to generate fully self‐powered systems is another option.^[^ ^375^ ^]^

This generation of implants is still in the preliminary stages of development, and only proof‐of‐concept tests have been carried out.^[^ ^16^ ^]^ Studies of these implants need to define the properties of implants with three autonomous features, e.g., self‐sensing, self‐powering and monitoring, and the efficiency of self‐aware implants in assessing the fusion process of the spine and energy harvesting from mechanical excitations needs to be defined. Furthermore, in vivo studies of these third generation autonomous implants are needed. Self‐aware implants can be considered a third generation owing to their multiple properties, such as self‐sensing, self‐monitoring and self‐powering through energy harvesting.

For scale‐up, e.g., self‐powering, self‐monitor and self‐sensing implants, the use of advanced fabrication technologies such as 3D printing may help design and scale up customized implants.^[^ ^376^ ^]^ Furthermore, the use of AI may also be helpful in this process. Prior to clinical studies, large animals should be used to define biocompatibility, safety, effectiveness, and durability. An appropriate power management module for the long‐term operation of nanogenerators used in implants and sensors with sensing capabilities should be optimized. In the example of self‐aware implants, the optimal mechanical and electrical performance of the implants should be proven.^[^ ^16^ ^]^ Similar to other generations (first and second), tests to demonstrate safety and efficacy are needed. The complexity of the third generation imposes more challenges on the type and number of tests to generate the data required to approve safety and efficacy. In addition, the models required to demonstrate the function of the 3^rd^ generation are more demanding.

The multifunctional metamaterials (third generation), which act as their own sensors, recording and generating their own power, have already been developed;^[^ ^16^ ^]^ moreover, the nanogenerator eliminates the need for a separate powering source. A tiny chip is embedded in self‐aware implants, which records data about the pressure in the spinal cage as an indicator of the healing process. To date, no in vitro, in vivo or clinical studies have been reported.

### 3.4 Fourth Generation (Tissue Mimic)

Fourth‐generation automated implants can be defined as implants with multiple features. These implants incorporate many capabilities to make them so close in mimicking native tissues (tissue‐like mimics). This type of implant that combines four automation properties does not currently exist, but it is expected to be the next step in the path of development of automated implants.

## 4 Applications

The autonomous implants discussed above can be investigated for various applications, such as bone regeneration,^[^ ^27^ ^]^ the bone healing process,^[^ ^284^ ^]^ wound healing^[^ ^162^ ^]^ and drug delivery.^[^ ^115^, ^118^, ^241^ ^]^ In addition to the applications tested thus far in animals, there are also other potential applications (**Table** **3**).

**Table 3:** Applications of different autonomous implants and their clinical outcomes.

Tissue/organ	Implant	Actual application	Potential application	Refs.
Bone	Self‐aware implant	Concept level: Interbody fusion cage prototype for detecting bone healing process using voltage signals via built‐in contact‐electrification mechanisms Test: synthetic spine models	Diagnosing bone healing process using voltage signals	
	Self‐powered implant	Self‐powered stimulator‐in vivo studies	Bone fracture and bone remodeling after bone transplantation	[284]
	Self‐actuating implant	Concept level: Bioinduced variable stiffness actuators with preprogrammed shapes that can change properties and grow own bone	Treatment of bone fracture and bone regeneration	[27]
	Self‐activating implant	Self‐activating coating for drug‐release of molecules (in vitro studies).	Antibacterial dental implants and improved bone regeneration.	[241]
	Self‐ activating implant	Self‐activating multifunctional hydrogel coating. (in vitro and in vivo studies)	Anti‐tumor therapy, antibacterial surface for implants and improve bone regeneration	[245]
CVS	Self‐aware implant	Concept level‐ meta‐tribomaterial nanogenerators with energy harvesting and sensing functionalities	Designing artificial materials for cardiovascular stents and shock absorbers	[66]
	Self‐healing	Scaffolds‐ In vitro *and vivo studies*	Wound healing, neural regeneration in the brain after ischemic stroke	[115, 118]
ENT (cochlear implant)	Self‐aware implant	Concept Level: Spiking neural network	Auditory system	
	Self‐healing	Hydrogels, *ex vivo* studies	Voice recovery	[28]
Gastrointestinal tract	Self‐orienting system	In vivo studies‐	Drug release capsule	[238]
	Self‐regeneration	Engineered living hydrogels, in vitro, in vivo studies	Mucosal wound healing	[162]
Tissue engineering and wound healing	Self‐forming implants	Stress‐induced rolling membranes, in vitro studies,	Artificial blood vessels and tubular structure in and tissue	
	Self‐powered	Self‐powered dressing, in vitro and in vivo studies	Wound healing, promising therapeutic platform for pathological disorders such as venous ulcers, ischemic wounds and keloid scarring	[290]
Ophthalmology	Self‐align implants	Intraocular implants have been shown to self‐align by hydrostatic pressure (in vivo studies)	The features of self‐aligning induce better outcomes in terms of intraocular stability of lens or intraocular devices	[251]

## 5 Current Challenges and Future Perspectives

### 5.1 Current Challenges

One of the major challenges facing all types of implants in the body is the immune body reaction, which affects the function and durability of the implant. Although various approaches to control these reactions and engineer immune responses have been explored,^[^ ^377^ ^]^ this remains a challenge that must be considered when designing autonomous implants in the future. The design of such implants should consider the integration of technologies that can modulate the immune response, such as surface patenting, the release of molecules or the integration of cellular and cell components into these implants.^[^ ^378^ ^]^ Another important challenge for any implant, including smart and autonomous implants, is the risk of implant‐related infections.^[^ ^379^, ^380^ ^]^ Recent advances in the fabrication of novel materials that have antimicrobial properties and can prevent biofilm formation should be considered in the design of autonomous implants. Smart bioresponsive materials that can turn on these antimicrobial mechanisms upon exposure to microorganisms by releasing drugs or changing their properties^[^ ^380^, ^381^ ^]^ should be explored and integrated. Other challenges that face implants in general include corrosion,^[^ ^382^ ^]^ allergic reactions,^[^ ^383^, ^384^ ^]^ and hypersensitivity reactions.^[^ ^385^ ^]^ In addition, there are challenges that can be more specific to autonomous implants. These include long‐term stability, continuous power generation and sensing capabilities, which are discussed in the following subsections. Self‐aware implants have a complex system with self‐powering and sensing units that require advanced engineering with multiple steps, including prototyping, testing and validation. The whole process can be time consuming and may create difficulty for the transition from the laboratory scale to the commercial scale.

To date, studies related to autonomous implants are limited. When investigated in vivo, small animals have been used, making it difficult to anticipate the outcome in larger animals and in clinical studies. Existing self‐powering implants have several challenging issues that need to be addressed before they can be promoted into next‐generation multifunctional autonomous implants to increase their market penetration. For example, most self‐powered implants depend on a single energy harvesting source, which limits their utilization for many applications. The integration of a system that depends on multiple power generation sources also has limitations due to its limited size, efficiency, capacity, working conditions, etc. The ability of energy harvesters to supply sufficient and continuous energy remains a major challenge in self‐powering implants. There is no proper power management or prototype that controls self‐powering and sensing implants with time management, and the duration of supply and life of the powering unit is not well reported. Embedding power harvesters, electronics, sensors and other units increases the size of the implant, which makes it difficult to implant the unit in the body. There are also cytotoxic and genotoxic risks related to the degradation or failure of self‐powering units, or their components are another major issue to consider for these implants. Interfunctional coordination between tissues, implants and inner parts remains a challenge that needs further research. The flexibility of the self‐powering unit and its components, adhesion to the body, biosafety and degradability are other crucial parameters that need more attention in future studies.

In the case of self‐healing implants, the healing ability decreases with time, and improvements in the mechanical properties are needed.^[^ ^386^ ^]^ Identifying appropriate materials for producing self‐healing implants that are associated with lower rates of wear and degradation is also a practical challenge. The potential side effects and long‐term stability of self‐healing materials are uncertain and may lead to inflammatory reactions during the healing process.^[^ ^387^ ^]^ The biocompatibility of implants remains a crucial concern, e.g., the self‐healing properties of implants created by employing coatings that provide both biocompatibility and self‐healing have been explored.^[^ ^105^, ^106^, ^107^, ^108^, ^109^ ^]^ As discussed, coating metallic implants with a layer that has self‐healing properties will help to repair defects autonomously and prolong the lifetime of the implant.^[^ ^105^, ^106^, ^107^, ^108^, ^109^ ^]^ Preserving self‐healing implants loaded with living cells is another challenge for large‐scale clinical application.

The fabrication of autonomous implants involves a variety of technical, safety, financial and privacy difficulties that must be overcome before they can be on the global market. For example, the fabrication of miniature‐sized sensors (to monitor, diagnose and detect) and nanogenerators that are integrated with the implant is a major challenge, as the transmitters, converters, detectors and other components have to be fixed in a limited area. Another crucial concern is the design of specific implant geometries, dimensions and angles that need to be adjusted with the hard or soft bones. The time needed to construct these implants and the high cost may be due to the patient‐specific design. The coating of implants with self‐healing materials is another challenge, as these materials need to adhere to the implant and be sustained for a long period of time. The reproducibility of design is another challenge, and safe procedures need to be undertaken to produce complex structures of implants. Challenges related to the manufacturing of self‐healing materials include those related to the inclusion of healing agents, the carrier for the healing agent, the integration with reinforcement, and the evaluation of healing efficacy.^[^ ^388^ ^]^

Challenges related to the characterization of autonomous implants include difficulty in characterizing different functionalities in the same implant at the same time, e.g., a 3^rd^ generation implant with self‐aware, self‐powering and self‐actuation implants poses a special challenge for characterization. Available standard tests may be not available. The tools needed to test these multifunctional implants may not always be efficient. There may be a need to develop new tools for defining the properties and testing such autonomous implant efficacy. Bringing these autonomous implants to wider implications is faced by the availability of enough strong evidence of their superiority over conventional implants. In addition, the price of an implant is likely to increase; hence, maintaining a balance would be necessary before it could be available commercially. All the components of the implants have to be sterilized, and the same sterilization protocol may not be suitable for all the components. Some materials may decompose or undergo phase changes during the sterilization process.^[^ ^389^ ^]^

### 5.2 Future Perspectives

Future research should focus on issues such as long‐term in vivo studies, advanced power sources, multiple sensor systems, and enhancing the biocompatibility of devices. Research pertaining to in vivo testing should focus on testing these implants in large animal models. In addition, scalability, cost‐effectiveness, multifunctionality and commercial availability should be the focus of future studies. The risks for self‐powering implants can be minimized if the implant is encapsulated with biocompatible materials, as well as by reducing the size of the inner components to form a miniaturized structure that can easily be implanted in the body. Strategies for prohibiting infections in self‐healing implants include inhibiting biofilm formation via surface coating,^[^ ^390^, ^391^ ^]^ the functionalization of surfaces with silver nanoparticles,^[^ ^392^ ^]^ mesoporous silica^[^ ^393^ ^]^ or natural compounds,^[^ ^394^ ^]^ the development of antiadhesive medical surfaces, and the immobilization of antimicrobial agents or antibiotics.^[^ ^380^, ^381^ ^]^ Advanced coating techniques are needed to increase the degree of implant‐tissue integration. Moreover, natural polysaccharides, proteins, growth factors and biomolecules can be utilized as coating adhesives; in particular, polymers with a high degree of biocompatibility and biodegradability offer substantial value. Integrating wireless technology with implants can offer additional advantages to implants where the sensors embedded in the implant can monitor and communicate to provide real‐time conditions during the healing process.

**Figure 10:**
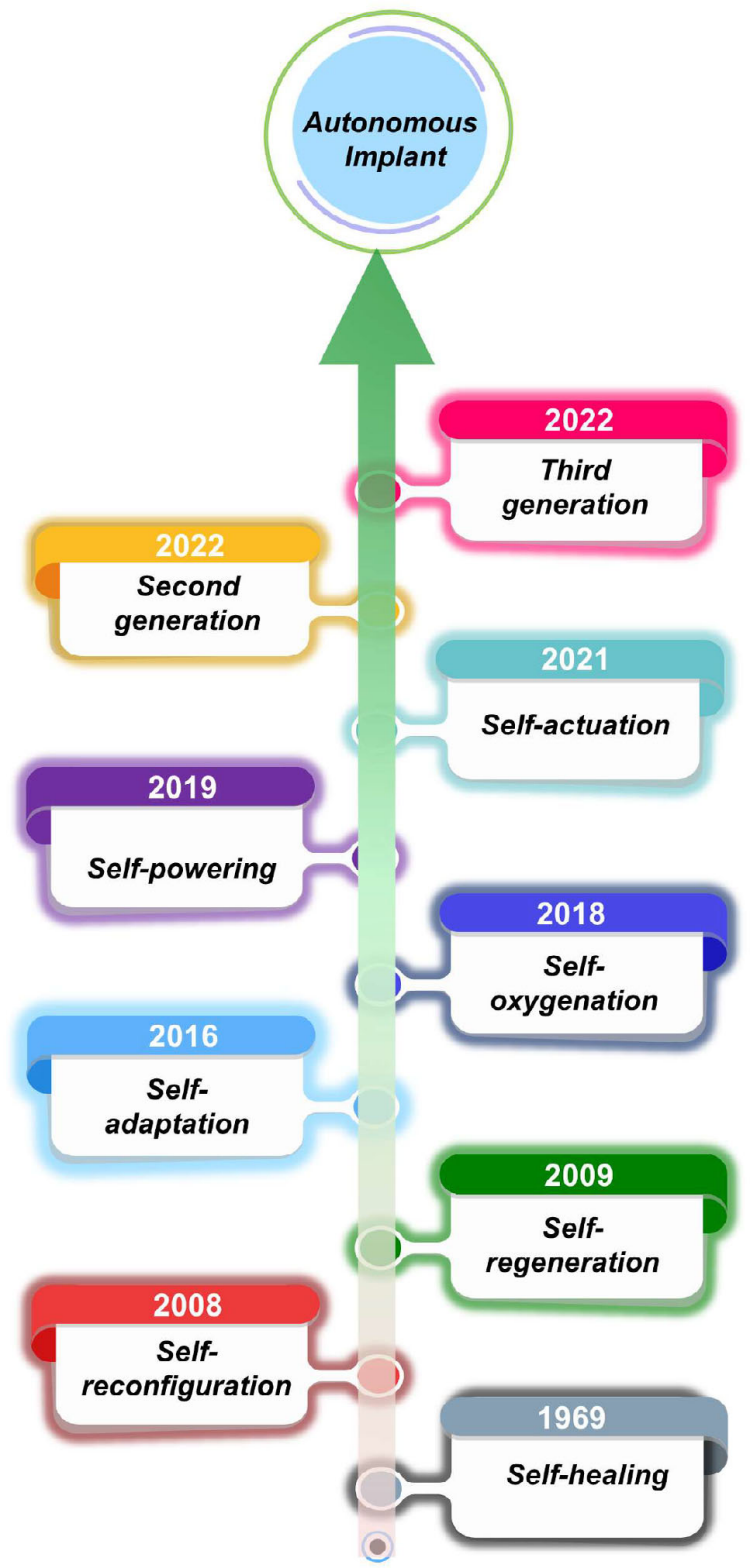
Roadmap of research activities toward the development of autonomous implants.

Research aiming to develop tissue‐mimic autonomous implants is evolving through the developments seen in individual lines required for individual autonomy characteristics, such as developing self‐forming, self‐regenerating, self‐healing and self‐powering properties (**Figure** **10**). The possibility of integrating these characteristics into specific implants on the basis of their target application and utilization is expected to be explored in the future. These autonomous implants will be independent, as they will sense changes early and undertake the necessary actuation required to address these changes, such as self‐healing or releasing therapeutics alerting doctors and patients, and may also receive instructions via AI in the future to take specific actions (to help or replace externally applied interventions that doctors have to carry out today). They perform multiple functions, such as self‐awareness, which can provide the required mechanical support; self‐power via nanogenerators; and self‐sensing and monitoring, which provide information to doctors through data generated in the implant. Self‐sensing allows us to detect any changes in implants and in the healing process of surrounding tissues or complications that may arise, such as infection, and will be able to alert us so that interventions can be instituted before damage becomes irreversible. Moreover, real‐time data from implanted sensors can provide vital information about implant integration into surrounding tissues, and both healthcare personnel and patients can become informed of the status of the implant. Self‐healing and regeneration can further enhance functionality, which can create an atmosphere to heal nearby tissues and regenerate damaged bones or tissues. Self‐actuation can monitor chemical or physical changes and trigger the release of cells, drugs, antibiotics, etc.

In early generations, autonomous implants, such as self‐aware, sensing implants, should be able to report any aberrations that normally occur in their body, function or surroundings to doctors or nurses. At later stages, when these autonomous implants become adopted in healthcare and patients are educated about them, patients may be able to follow them through their electronic systems, including cell phones, and report them to health care providers to seek appropriate interventions. At these stages, the implants should become more independent of external intervention and be able to recognize things that are wrong and implement appropriate action, i.e., when they become truly more like native tissue in the next 40–50 years.

Autonomous implants are the beginning of a new technological revolution that will also benefit and integrate other technologies to improve the quality of implants and their diagnosis, detection of structure, reliability and safety. The integration of technologies such as computer tomography^[^ ^395^ ^]^ and AI^[^ ^396^ ^]^ facilitates 3D visualization of anatomical structures and improves radiographic diagnosis, precision, and efficiency of implants. For example, computed tomography has been utilized to analyze trabecular bone structure around dental implants,^[^ ^395^, ^397^ ^]^ analyzing bone remodeling around percutaneous osseointegrated implants,^[^ ^398^ ^]^ etc. Other examples include AI‐driven image analysis and deep learning algorithms, which increase the precision of dental implants, and AI‐driven neural implants, which are effective in tracking brain activity, such as the progression of Alzheimer's disease.^[^ ^399^ ^]^ AI algorithms for total joint arthroplasty analysis have shown promising results in terms of identification, implant failure and measurement of implant dimensions.^[^ ^396^ ^]^ Technological advancements enable the manipulation of implant surfaces and designs to reduce peripheral damage to host tissues and provide efficient pharmaceutical remedies, e.g., in orthopedic oncology,^[^ ^400^ ^]^ and dental departments.^[^ ^401^ ^]^ The integration of nanoengineered strategies can help design implants with structural stability, pore sizes appropriate for cell migration, mechanical support and drug release through the implants.^[^ ^402^ ^]^ Self‐powered sensors and advanced nanogenerators can also play a major role in designing these autonomous implants.^[^ ^17^, ^403^, ^404^ ^]^

Wireless network approaches are advancing, and the implants use radio frequency,^[^ ^405^ ^]^ optical^[^ ^406^ ^]^ or ultrasound^[^ ^407^, ^408^ ^]^ technology to wirelessly communicate. The distribution network of these implants may help us regulate autonomous functions,^[^ ^409^, ^410^ ^]^ such as closed‐look sensory feedback,^[^ ^411^ ^]^ stimulation of peripheral nerves,^[^ ^412^ ^]^ adaptive pain management,^[^ ^413^ ^]^ and the management of diabetes.^[^ ^414^ ^]^ However, these methods have low‐efficiency transmission through biological tissues and are difficult to miniaturize. Existing implants with wireless communication systems rely on indirect signal transmission via battery‐powered devices placed outside the body,^[^ ^414^, ^415^, ^416^ ^]^ which need to be periodically recharged. Recently, metamaterials have been broadly applied in wireless technology for communication,^[^ ^417^, ^418^ ^]^ sensing,^[^ ^419^ ^]^ and Wi‐Fi power transfer.^[^ ^420^ ^]^

There is a significant need to develop autonomous implants with multifunctional features employing an advanced and sustained approach through the combination of all the concepts and technologies presented here. The key concept in designing implants lies in their safety and effectiveness for the intended purpose. The current literature on various self‐functioning implants in different sections has widened the thought process of integrating various functionalities and properties (such as minimum size and weight, low power consumption, sterilizability, low toxicity, and economic aspects) in a single autonomous system. Future research can pave the way for autonomous implants with self‐monitoring and detection techniques, where the patient can instantly diagnose his medical condition for quick action. Extensive preclinical studies are needed on these autonomous implants in accordance with the relevant international standards and national regulations before they can be placed under diverse situations. Moreover, advanced techniques such as digital planning, 3D printing and surface modification of implants will increase the feasibility of implant placement and monitoring, resulting in better outcomes. Future implant design may focus on osteoimmunology, with emphasis from *immune evasion* to *immune reprogramming*.^[^ ^382^ ^]^

## 6 Conclusions

Important research has been carried out to develop and characterize or optimize self‐healing, self‐aware, self‐regenerating, self‐actuating, self‐powering and self‐oxygenated autonomous implants. Integrating these capabilities will enable the development of implants in the future that can be autonomous in the sense that they can sense their surroundings, communicate, heal themselves and remodel. Autonomous implants have the potential to have multifunctional features through the integration of multiple components, but more research is needed to optimize implant design, materials, interactions with the human body and long‐term utilization. The reliability of these implants for multifunctional purposes should be evaluated before their use in clinical practice. The studies carried out for individual self‐working implants seem to have advanced substantially, possibly because they are self‐aware, self‐healing, and self‐powering; however, when two self‐featured implants are combined into one, the properties, stability, mechanical properties, and compatibility completely change, which needs further research. The development of an autonomous implant for targeted application will require collaborative and interdisciplinary research in areas such as microbiology, biotechnology, chemistry, biomaterials, endoscopically assisted examinations, evaluation for treatments, and multicenter studies so that preventive strategies can be designed. In some cases, the implants need to stay in the body for a longer duration or rest; therefore, designing autonomous implants with multiple functionalities is crucial to avoid multiple surgeries. Many aspects should be considered; for example, self‐powered sensors with long‐term stability must be designed that can withstand multiple stretching cycles, and reliable energy harvesters are necessary to create advanced strategies that can generate power, as long as they are implanted inside the body. Furthermore, proper implant shape, size and physical properties are needed to avoid damage to the surrounding tissues. We need to move forward with next‐generation innovations with techniques such as 3D printing, artificial intelligence, gene therapy and nanotechnology, which will help to design much‐advanced autonomous implants with multifunctionalities.

## Conflict of Interest

The authors declare no conflict of interest.

## Data Availability

The data described in the article are available at http://zenodo.org/records/15826453. The authors would appreciate if other researchers could benefit from our literature and results. This will foster discussions and collaboration among scientists worldwide.
